# Atomically Precise Cluster Cocatalysts: Missing Link toward Heterogenized Photocatalytic Systems

**DOI:** 10.1021/acsnano.5c15286

**Published:** 2025-12-19

**Authors:** Stephen Nagaraju Myakala, Alexey Cherevan

**Affiliations:** Institute of Materials Chemistry, TU Wien, Getreidemarkt 9/BC/02, Vienna 1060, Austria

**Keywords:** hydrogen evolution reaction, water splitting, CO_2_ reduction, thiometalate, polyoxometalate, nanocluster, secondary building unit, cocatalyst, surface anchoring, structure−activity relationship

## Abstract

This review critically examines the emerging role of atomically precise clusters – oxide, sulfide, and metallic in nature – as transformative cocatalysts for photocatalytic solar fuel production. Unlike structurally imprecise nanoparticles with ill-defined active sites or molecular organometallic complexes with limited stability, these clusters offer atomically resolved structures that bridge homogeneous precision with heterogeneous robustness. Their well-defined multinuclear architectures enable precise control over their active sites, facilitating systematic studies of structure–activity relationships and mechanistic insights critical to rational catalyst design. Compared to contemporary cocatalytic systems, these clusters offer tunable compositions and structures, enhanced stability on the surface, and the capacity to engage in multielectron redox processes. We review recent experimental developments, discuss strategies of their surface-anchoring, and highlight mechanistic insights provided by their use. Finally, we critically evaluate current challenges and propose future research directions to unlock the full potential of cluster-based cocatalysts as a tool for purposeful engineering of active and selective photocatalysts for light-driven solar fuel generation.

## Graphical abstract

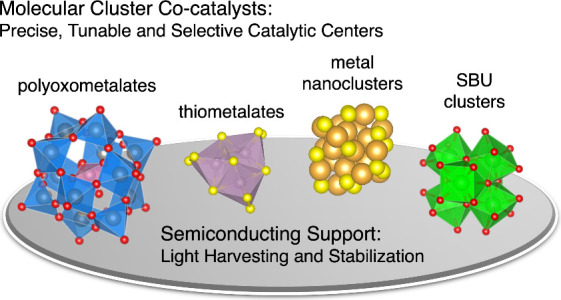

## 1 Introduction

Catalysis has fundamentally transformed the human society over the past 150 years, enabling the production of abundant energy, food, and advanced materials that underpin modern life. It has been instrumental in driving industrialization and facilitating economic growth, improving living standards across the globe. However, this development has come at a steep environmental cost and now contributes heavily to the growing tensions around natural resources.^1,2^ In response, the development of sustainable, carbon-neutral energy conversion technologies has become an urgent priority. Photocatalysis, which harnesses solar energy to drive chemical transformations, offers a promising pathway toward renewable fuel production and environmentally friendly chemical processes.^3^ In a typical photocatalytic process, absorption of light by a semiconductor photocatalyst generates free-moving charge carriers, which then migrate to the surface to drive redox reactions by transferring electrons and holes to appropriate acceptor and donor molecules. By directly utilizing sunlight, photocatalytic systems can potentially provide decentralized, scalable solutions for converting abundant feedstocks like water and biomass into energy-rich fuels such as hydrogen.^4–6^

Despite significant progress achieved in the field over the past 50 years – including the development of a photocatalyst that enables quantum efficiency of 96%,^7^ demonstration of solar H_2_ production on a 100 m^2^ scale and with solar-to-hydrogen (STH) conversion efficiency of more than 9%^8^ – the widespread implementation of photocatalytic technologies still faces persistent challenges. Current photocatalysts often suffer from limited activity, poor selectivity, and insufficient long-term stability under operational conditions. Moreover, the complex interplay of light absorption, charge separation, and surface reaction dynamics makes the rational design of efficient photocatalytic systems particularly demanding.^9^ Recent advances in materials chemistry, driven by an atomistic understanding of catalytic processes and increasingly supported by artificial intelligence and machine learning tools,^10^ offer new opportunities to overcome these limitations. By integrating predictive modeling, high-throughput screening, and data-driven materials discovery,^11–13^ it is possible to accelerate the development of tailored photocatalysts with improved performance and stability. One crucial milestone to this endeavor, however, is the ability to construct and study well-defined model systems that allow precise control over composition, structure, and active site properties, providing essential insights into the structure–activity–stability relationships that govern photocatalytic reactions.

Cocatalysts are at the heart of many photocatalytic systems to enable a selective and efficient conversion.^14,15^ While the photocatalytic support absorbs light and generates charge carriers, cocatalysts typically accelerate charge separation by extracting photoexcited carriers and lower activation barriers by providing active sites for the desired chemical transformation. Most of the cocatalytic systems are based on small inorganic nanoparticles (NPs) such as in the exemplary cases of RuO_*x*_,^16,17^ Pt,^18,19^ core–shell Rh/Cr_2_O_3_,^20^ NiO_*x*_,^21^ CoPi,^22,23^ and PtZn intermetallic^24^ and Au_*x*_Ag_1–*x*_ alloy.^25^ While many of these cocatalysts are successful at realizing the desired conversion – be it oxygen evolution reaction (OER), hydrogen evolution reaction (HER), or CO_2_ reduction reaction (CO_2_RR) – such inorganic NPs are inherently poorly defined in their structure and composition given the natural spread of their particle size, shape, facets, and surface chemistry.^26^ The inability to precisely control and manipulate their active sites makes it virtually impossible to capitalize on the wide experimental evidence and realize the purposeful design of next-generation cocatalysts.^27^ The only rational way to escape the established trial-and-error approach in designing advanced cocatalysts is to switch to employing cocatalytic systems that are molecular in nature.

This idea has long been followed in the field, with numerous reviews written on the topics of heterogenization,^28,29^ surface-anchoring,^30,31^ or bridging^32–34^ homogeneous and heterogeneous branches: all pretty much talking about the same conceptual thing. The use of a structurally and compositionally well-defined (molecular) cocatalyst is a real game changer as it allows us to study and rationalize the structure–activity relationship in unprecedented detail. Several research directions that rely on both bottom-up and top-down strategies can be distinguished. One of them aims to anchor molecular organometallic complexes – known for their excellent catalytic performance in the homogeneous phase – to the surface of solid-state inorganic photoactive substrates. While many groups actively work on these grounds,^35–38^ the main drawback of this approach is related to the insufficient stability of the molecular redox systems, which remains an issue under harsh oxidative conditions of a photocatalytic process. The other strategy relies on the generation of single- and few-atom cocatalysts and takes its inspiration from earlier works on single-site heterogeneous catalysis.^39,40^ Owing to the initial developments by the group of Yamashita,^41,42^ many prominent examples of noble-metal^43–46^ and transition-metal-based^47,48^ single-site photocatalysts for water splitting and CO_2_RR have been reported in the past years. The ongoing work shows that not only turnover frequency (TOF) numbers of isolated single metal sites can be enhanced drastically compared to the case of common bulky cocatalyst NPs, but also the reaction selectivity can be controlled.^49^

One underexplored direction, which we believe stands out and has the ability to fill the gap in this exploration, is the use of atomically defined molecular clusters as cocatalysts. Compared with organometallic complexes, their all-inorganic cores can offer rich redox chemistry and superior chemical stability, particularly in aqueous and oxidative environments, which are central to solar fuel production. Compared to single-atom catalysts, their well-defined molecular structures and tunable chemical properties can enable systematic studies of structure–activity relationships and mechanistic pathways, providing a foundation for rational, predictive catalyst design.^50^ On top of this, the multinuclear nature of such clusters can be ideal to provide a platform and explore multimetallic assemblies and the concept of dual- and triple-site catalysts.^51^ This review explores the use of all-inorganic molecular clusters as cocatalysts in a range of photocatalytic applications. First, we review and classify the types of clusters and discuss their generally relevant characteristics. This is followed by an overview of the experimental work done so far, where we also highlight the promise behind this unique span of compounds and their use in photocatalytic applications. We conclude this review with a critical evaluation of the current state of the art and provide potential directions for further research toward the exploration and development of cluster-based photocatalytic systems.

## 2 Classification

Various classes of cocatalysts that have been used to construct photocatalytic systems ranging from atomic or molecular all the way to particulate or bulk (Figure 1, top).^52–55^ These two extremes can be classified as purely homogeneous and heterogeneous types. Driven by the idea to close the gap between these two branches, both communities have been moving toward each other over the past decades. As such, typical cocatalyst nanoparticles have been downsized all the way to quantum dot sizes^56–58^ and molecular monolayers,^59^ whereas organometallic chemistry has been used as a precursor to construct well-defined multimetallic assemblies on surfaces.^60^

**Figure 1:**
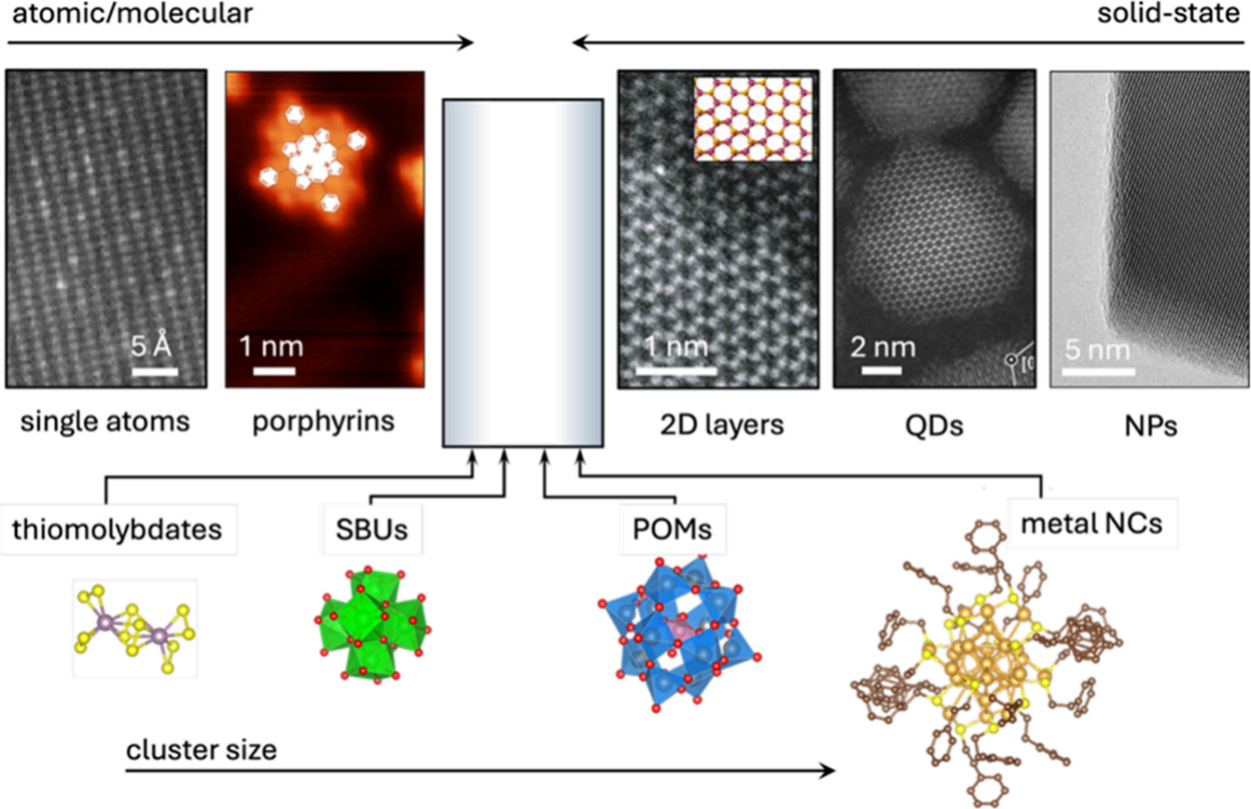
(top) Merger of molecular and solid-state cocatalyst. (bottom) Relative size distribution of the different classes of cluster cocatalysts discussed in this review, shown at-scale based on their CIF files and using VESTA software. From left to right: Single atoms, adapted with permission from ref 52. Porphyrins, adapted with permission from ref 53. Copyright 2014, American Chemical Society. 2D layers, adapted with permission from ref 54. QDs, adapted with permission from ref 55. Copyright 2014, American Chemical Society.

Copyright 2024, Springer Nature.

Copyright 2018, Springer Nature.

Still, the gap between an (inorganic) nanoparticle and an organometallic complex remains wide, with just a few compound classes able to effectively bridge it. These include well-defined metal thiometalate and polyoxometalate (POMs) clusters, metal nanoclusters (NCs), and unique secondary building units (SBUs) stabilized inside metal–organic frameworks (MOFs). Figure 1 (bottom) contains an at-scale schematic illustration of these types, while Table 1 summarizes their general features. POMs and thiometalates can be seen as metal oxygen (oxide) and metal sulfur (sulfide) clusters, respectively. The size of POM clusters is typically around 1 nm, but much larger POM assemblies up to 5 nm are known to exist^61^; thiometalates, on the other hand, are much smaller with bi- and trinuclear transition-metal-based cores dominating their structural variety. Metal nanoclusters typically feature purely metallic cores – such as in Au_25_ and Au_38_ NCs stabilized by a monolayer of organic ligands – whose composition and structure can be tuned with atomic precision. Their size ranges from subnm to approximately 2 nm. Secondary building units of MOFs feature a wide variety of compositions and structures and typically contain between 2 and 8 metallic centers connected by O, S, or N-binding bridging ligands; however, infinite chain-like or layered SBUs are also known.^62,63^ Their size thus also lies in a subnanometer range, which – similar for the case of other clusters – turns these strictly inorganic systems into molecular systems with no bulk but only (or mostly) surface, enabling high atom utilization efficiency, predictive modeling, and data-driven materials discovery.

**Table 1:** Overview Table Comparing the Four Major Cluster Cocatalyst Classes Discussed in This Review

**cluster type**	**typical size**	**core structure**	**anchoring strategy**	**primary applications**	**stability concerns**
thiometalates	0.5–1 nm	Mo, W chalcogenide (S); O, Se and organic ligands possible	covalent/electrostatic on oxides, sulfides, nitrides, polymers	HER	sulfide loss or exchange; cluster disintegration or oligomerization
polyoxometalates (POMs)	0.5–5 nm	W, Mo, V, Nb oxides; incorporation or sandwiching of noble- and transition metals; organic ligands possible	covalent/electrostatic, linker-mediated on inorganic and organic semiconductors; encapsulation or embedment into MOFs	HER, OER, CO_2_RR	pH-dependent aggregation or dissolution; reduction/decomposition under light
secondary building unites (SBUs)	0.5–2 nm	transition-metal-oxo clusters; 1D and 2D SBUs possible; variety of ligands possible	embedded in MOF; covalently or electrostatically surface-grafting	HER, OER, CO_2_RR	framework degradation under reactive or irradiation conditions; ligand exchange
metal nanoclusters (NCs)	1–2 nm	Metallic (e.g., Au, Pt, Ag, Cu); organic ligand shell	ligand shell exchange; direct deposition; polymer encapsulation	HER, CO_2_RR	ligand leaching, core aggregation; surface restructuring

These four classes of molecular cluster cocatalysts offer unparalleled opportunities for advancing our understanding of photocatalytic processes. Their well-defined, atomically precise structures allow for the elucidation of catalytic mechanisms at an unprecedented level of detail. The structural precision and compositional variability enable the pinpointing of active sites and tracking of electron transfer pathways, providing critical insights into how these clusters engage into redox reactions. By studying these mechanisms, we can uncover the fundamental principles governing photocatalysis, paving the way for the design of more efficient and selective photocatalytic systems.

## 3 Types of Clusters and Their Roles in Photocatalysis

Section 3 surveys four principal classes of atomically precise cluster cocatalysts – thiometalates, POMs, SBUs, and metal NCs – as platforms for photocatalytic solar fuel generation. Each class is distinguished by unique core structures, ranging from chalcogen-bridged transition metals to discrete metal-oxo assemblies, highly tunable inorganic SBUs, and metallic quantum-confined clusters. These systems offer advantages such as precise control over active site structure, tunable electronic and redox properties, and the capacity for multielectron redox catalysis. The following subchapters provide a broad overview on each of the cluster types and discuss their prior use as cocatalysts. We highlight the most relevant examples of their implementation and dive into the mechanistic and structural insights enabled by their well-defined molecular nature.

### 3.1 Thiomolybdates

Thiomolybdates are a unique class of all-inorganic molecular clusters composed of molybdenum centers coordinated by sulfur ligands. Landmark work by Müller and colleagues in the 1970s led to the synthesis and characterization of prototype species like [Mo_2_S_12_]^2–^ and [Mo_3_S_13_]^2–^, obtained via reduction of molybdenum­(VI) precursors in polysulfide-rich aqueous solutions.^64,65^ Despite their early synthesis, thiomolybdate clusters, particularly [Mo_3_S_13_]^2–^ and [Mo_3_S_4_]^4+^ ions, have only recently emerged as highly promising molecular catalysts toward HER due to their sulfur-rich, bioinspired structures that mimic the active sites of bulk MoS_2_ (Figure 2a).^66^ The first report to uncover the catalytic potential of a surface-anchored thiomolybdate cluster toward electrochemical HER appeared in 2008.^67^ This work was followed by a number of in-depth studies to reveal the role of the electrochemical support,^68^ cluster nuclearity,^69^ and composition,^70^ as well as the type of its ligands^71^ on the resulting electrochemical performance, with outstanding activities (high current densities and low overpotentials) commending thiomolybdates for this application (Figure 2b). Surface-sensitive techniques like scanning tunneling microscopy (STM) and X-ray photoelectron spectroscopy (XPS) were used to confirm successful anchoring and follow structural evolution of the clusters upon heterogenization. These supported thiomolybdate catalysts could achieve onset potentials as low as −0.2 V versus reversible hydrogen electrode (RHE) and competitive turnover frequencies.

**Figure 2:**
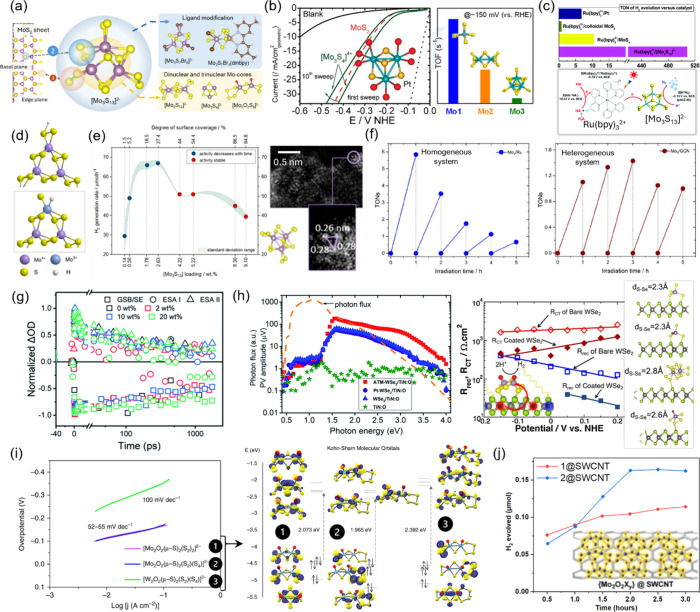
(a) Schematic illustration showing the edge of an exfoliated MoS_2_ sheet (left), [Mo_3_S_13_]^2–^ cluster (center), and possibilities for MoS_*x*_ cluster nuclearity and ligand modulation (right). (b) Polarization curves (5 mV/s scan rate) benchmarking xc72-supported [Mo_3_S_4_]^4+^, Pt and MoS_2_ toward HER. Inset: molecular structure of [Mo_3_S_4_]^4+^ core surrounded by O (red) of the aqua ligands (left) and TOFs obtained using different nuclearity thiomolybdates (Mo_1_: [MoS_4_]^2–^, Mo_2_: [Mo_2_S_12_]^2–^, Mo_3_: [Mo_3_S_13_]^2–^) (right). Adapted with permission from refs 67 and 69. Copyright 2008, American Chemical Society. (c) TONs comparing the photocatalytic H_2_ performance of Pt, MoS_2_, and [Mo_3_S_13_]^2–^ (top) and the proposed photocatalytic reaction mechanism (bottom). (d) Schematic showing two proposed HER mechanisms on thiomolybdate clusters involving an S-edge site (top) and Mo-hydride formation (bottom). (e) Photocatalytic HER rates as a function of [Mo_3_S_13_]^2–^ loading on TiO_2_ (left) and STEM image showing the {Mo_3_} core of the Mo_3_/TiO_2_ composite (right). Adapted with permission from ref 81. Copyright 2022, American Chemical Society. (f) Long-term HER performance comparing TONs of the [Mo_3_S_13_]^2–^ cluster under homogeneous (with [Ru­(bpy)_3_]^2+^) and heterogenized (with C_3_N_4_) conditions. (g) Fs-transient absorption decay kinetics of [Mo_3_S_13_]^2–^/C_3_N_4_ measured using 325 nm excitation in H_2_O. Adapted with permission from ref 85. Copyright 2022, Royal Society of Chemistry. (h) Surface photovoltage (TSPV) plotted as a function of the incident photon energy comparing pristine WSe_2_ and [Mo_3_S_13_]^2–^/WSe_2_ films (left, adapted with permission from ref 87. Copyright 2020, Royal Society of Chemistry) as well as charge transfer (*R*_CT_) and recombination resistances (*R*_rec_) drop upon [Mo_3_S_13_]^2–^ incorporation (extracted from impedance data measured under 450 nm illumination) along with the proposed atomic structures of the thiomolybdate/WSe_2_ interface. (i) Tafel slopes of W/Mo-oxysulfide clusters measured in 1 M H_2_SO_4_ (left) and their molecular orbital analysis (right). Adapted with permission from ref 71. Copyright 2019, Springer Nature. (j) Photocatalytic H_2_ values comparing [Mo_2_O_2_S_8_]^2–^ (1) and [Mo_2_O_2_Se_6_]^2–^ (2) on SWCNT.

Adapted with permission from ref 73. Copyright 2018, Royal Society of Chemistry.

Adapted with permission from ref 78. Copyright 2022, Springer Nature.

Adapted with permission from ref 84. Copyright 2024, Royal Society of Chemistry.

right, adapted with permission from ref 90, Copyright 2020, Elsevier

Adapted with permission from ref 92. Copyright 2025, Royal Society of Chemistry.

#### 3.1.1 Light-Driven Catalysis

Parallel to this development in the field of electrocatalysis, both [Mo_2_S_12_]^2–^ and [Mo_3_S_13_]^2–^ clusters have also been explored as catalysts in light-driven HER under homogeneous conditions in a combination with photosensitizers such as [Ru­(bpy)_3_]^2+^ and sacrificial electron donors.^72–74^ These fully molecular photosystems exhibited high initial activity, with reasonable TOFs (Figure 2c); however, a major limitation was found in their stability, with ligand exchange, agglomeration, and degradation in aqueous media leading to a gradual loss of performance. Despite this limited applicability, the molecular nature of thiomolybdates has been instrumental in allowing us to shed light on their catalytic sites and HER reaction mechanisms, revealing the importance of disulfide ligands (Figure 2d)^72,75,76^ and the possibility of Mo-mediated pathways.^77–79^

In line with the theme of this review article, thiomolybdates have more recently been applied as an HER cocatalyst to construct heterogeneous photosystems by exploring their anchoring onto oxide, polymeric, and porous photoactive supports. In an early pioneering work,^80^ [Mo_3_S_7_]^4+^ clusters have been supported onto the TiO_2_ surface but have been shown to rather act as a precatalyst that was converted into MoS_*x*_ species under turnover conditions. In a more recent study by Batool et al.,^81^ covalent binding of [Mo_3_S_13_]^2–^ onto TiO_2_ NPs was suggested following a self-assembly of the clusters onto the oxide surface in a monolayer fashion. XPS revealed the formation of Mo–O–Ti bonds at the molecular/inorganic interface offering a robust and irreversible attachment, whereas the resulting [Mo_3_S_13_]^2–^/TiO_2_ hybrid materials delivered HER rates comparable to those cocatalyzed by Pt NP benchmarks (Figure 2e). A similar result was next demonstrated for the binuclear [Mo_2_S_12_]^2–^,^82^ confirming promising cocatalytic function of thiomolybdates in light-driven catalysis on par with their excellent electrocatalytic performance.

#### 3.1.2 Advanced Supports

Several works have also explored the possibility of thiomolybdate heterogenization onto carbon nitrides (C_3_N_4_) taking advantage of their polymeric nature, photoresponse in the visible light range, and the possibility of controlling their surface charge. In one example, protonated C_3_N_4_ was used to electrostatically assemble anionic [Mo_3_S_13_]^2–^ clusters to enable high-performance visible light-driven HER.^83^ In another example, it was also demonstrated that immobilization of [Mo_3_S_13_]^2–^ onto graphitic C_3_N_4_ can lead to improved stability of the photosystem by benchmarking its performance against its homogeneous [Mo_3_S_13_]^2–^/[Ru­(bpy)_3_]^2+^ counterpart (Figure 2f).^84^ While some cluster leaching was observed over extended reaction periods, the remaining anchored catalysts (Mo_3_/C_3_N_4_) maintained substantial activity, whereas a continued deactivation was observed in the molecular photosystem (Mo_3_/Ru). In a complementary work, strong photoluminescence (PL) properties of supporting C_3_N_4_ allowed us to reveal the charge separation and transfer mechanism at the [Mo_3_S_13_]^2–^/C_3_N_4_ interface (Figure 2g).^85^ The authors reported that effective yet slow – on the ns-to-s scale – extraction of the electrons photoexcited in the C_3_N_4_ by the thiomolybdate takes place, which showcased the importance of the balanced cluster loading values (2 vs 20 wt %) in optimizing charge migration against charge trapping.

Motivated by earlier work on silicon photocathodes,^86^ thiomolybdate clusters have also been combined with transition-metal dichalcogenides (TMDs) aiming to enhance their photoelectrochemical performance. Bozheyev et al. reported that deposition of [Mo_3_S_13_]^2–^ onto polycrystalline WSe_2_ results in a dramatic 2-fold increase in its HER photocurrent density reaching record-breaking 5.6 mA cm^–2^.^87^ This effect was attributed to a strong cocatalytic effect of the thiomolybdate overlayer, which mitigates charge carrier recombination at edge states of the hexagonal WSe_2_ nanoflakes typical for the neat photoelectrode. Following this, several follow-up studies appeared pursuing the same [Mo_3_S_13_]^2–^/WSe_2_ heterojunction^88^ including a detailed study by transient surface photovoltage (TSPV) spectroscopy, which demonstrated effective separation of charge carriers and their transfer from WSe_2_ to the cluster sites (Figure 2h, left).^89^ One additional reason for the excellent performance of this [Mo_3_S_13_]^2–^/WSe_2_ couple was proposed by Chane-Ching et al.^90^ who coined the self-healing effect arising from structural similarity of thiomolybdates and TMDs. Confirmed by density functional theory (DFT) calculations, it suggests that oxo-thio complexes that coexist in the deposition and reaction solutions containing original thiomolybdate clusters can adsorb strongly onto point defects of the WSe_2_ surface, such as W or Se vacancies (Figure 2h, right). This adsorption passivates defects that would otherwise act as recombination centers for charge carriers, improving catalytic efficiency; it also leads to a strong covalent-like binding of the thiomolybdate clusters important to establish a strong molecular/solid-state interface, resulting in lowered charge transfer resistance (Figure 2h, middle). It is reasonable to assume that further control over the cluster structure, the type of its ligands and nuclearity can be used as a tool to optimize the deposition and performance of thiomolybdate cocatalyst combined with other photoactive semiconducting sulfides or selenides, similar to the case of recently reported [Mo_3_S_13_]^2–^/Sb_2_Se_3_.^91^

#### 3.1.3 Molecular Design

The use of thiomolybdate cocatalysts not only allows for high-performing photosystems with an activity level similar to that of noble-metal-based cocatalysts, but their unique molecular nature also enables gaining insights into the reaction mechanisms and the identification of potential active sites. Miras et al. for the first time investigated a structurally identical W-analogue of the binuclear [Mo_2_S_12_]^2–^ cluster, which allowed them to correlate metal identity to electronic structure of the cluster and its reactivity (Figure 2i).^71^ Extensive DFT calculations suggested that the unoccupied molecular orbitals of thiometalates are predominantly metal-based (W or Mo) so that a direct link between the metal type and the electronic structure governing reduction potentials and proton affinity can be established. Despite the fact that the HER mechanism proceeds via the protonation of ligands, the type of metal seems to define the relative affinity of different adsorption sites, be it terminal or bridging sulfides, as well as the energy of S–S bond cleavage upon its interaction with protons. In a similar manner, the impact of ligand structure was showcased when two unique oxysulfide and oxyselenide clusters: [Mo_2_O_2_S_8_]^2–^ and [Mo_2_O_2_Se_6_]^2–^ were noncovalently heterogenized onto single-walled carbon nanotubes (SWCNTs) to act as cocatalysts in electrochemical and photochemical HER (Figure 2j).^92^ Based on the terminal-chalcogen-ligand-centered HER mechanism proposed for Mo_2_ dimers earlier,^71^ the comparison of the two compounds allowed atomic-level insights into their ligand-dependent performance. The authors showed that [Mo_2_O_2_S_8_]^2–^ promotes faster H_2_ evolution kinetics and achieves lower HER overpotentials against [Mo_2_O_2_Se_6_]^2–^ under electrochemical conditions, suggesting superior catalytic property of S-based ligands. Despite this catalytic ranking, the Se-based cocatalyst cluster still showed superior photocatalytic HER (Figure 2j), which could be attributed to additional benefits of Se-based ligands in enhancing light harvesting. These examples demonstrate that molecular precision of such clusters allows direct attribution of mechanistic steps and thermodynamic driving forces to the specific metal and ligand type in the cluster core, which – in combination with structural tunability of thiomolybdates – can be used as tools to tune and optimize their reactivity or interaction with photoactive supports.

### 3.2 Polyoxometalates

Polyoxometalates (POMs) are a broad class of metal–oxygen clusters composed of early transition metals (most often Mo, W, V, and Nb) in their high oxidation state. Metal-oxo building blocks (commonly MO_6_ octahedra) often coexist with tetrahedrally coordinated heteroatoms (such as P or Si) and can be interconnected in a variety of ways resulting in many structural classes including most common Keggin, Wells–Dawson, Silverton, Waugh, Anderson, and Lindqvist (Figure 3a, right). Of interest to this review, POMs constitute another unique class of all-inorganic molecular clusters owing to their molecular nature coupled with their reversible electron storage capabilities, tunable electronic properties, and well-defined geometrical structures and composition.

**Figure 3:**
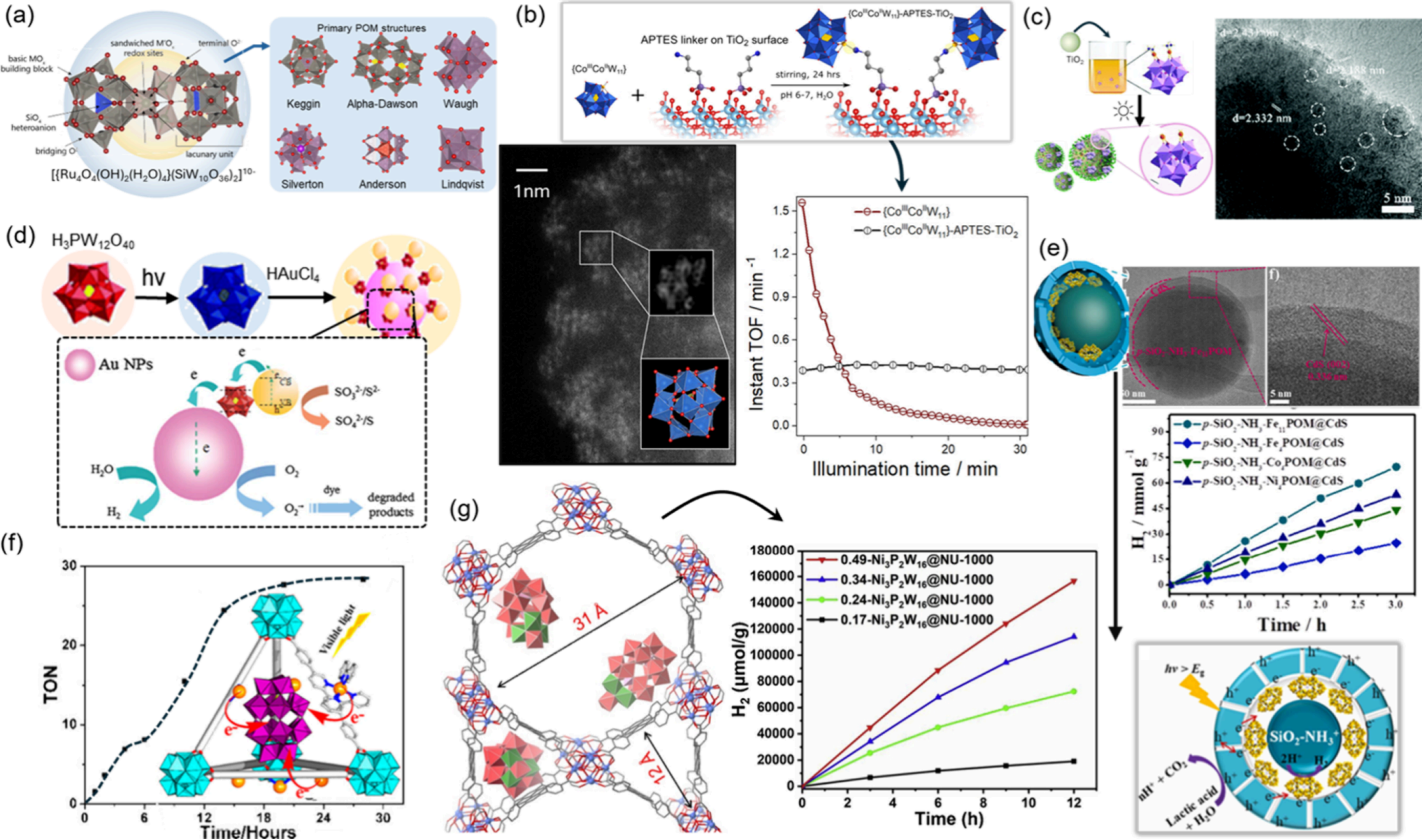
(a) Scheme showing a lacunary sandwich [{Ru_4_O_4_(OH)_2_(H_2_O)_4_}­(SiW_10_O_36_)_2_]^10–^ POM (left) along with common POM structures (right). (b) Schematic showing linking mode of a [Co^III^Co^II^(H_2_O)­W_11_O_39_]^7–^ (Co_2_W_11_) POM onto an APTES-functionalized TiO_2_ surface (top), STEM image resolving individual Co_2_W_11_ clusters on the surface (bottom left), and the instantaneous HER TOFs comparing its photocatalytic performance under homogeneous and heterogenized reaction conditions (bottom right). Adapted with permission from ref 109. Under CC by 4.0 Copyright 2022, American Chemical Society. (c) Schematic showing the synthesis of [{Pt­(NH_3_)_2_}_2_PW_11_O_39_]^3–^ (Pt_2_W_11_) POM anchored onto the TiO_2_ surface via photoreduction (left) and the corresponding TEM image of the composite (right). (d) Schematic showing synthesis of the Au-NP/[PW_12_O_40_]^3–^/CdS sandwich ternary structure along with the proposed photocatalytic HER mechanism. (e) TEM images showing the core–shell structure of [Fe_11_(OH)_2_(W_3_O_10_)_2_(α-SbW_9_O_33_)_6_]^27–^ (Fe_11_) POM encapsulation with a schematic representation (left) and its photocatalytic HER performance along with the reaction mechanism (bottom). (f) HER TONs of an encapsulated [P_2_W_18_O_62_]^6–^ (P_2_W_18_) POM inside a UiO-type MOF using [Ru­(bpy)_3_]^2+^ as a light sensitizer. Adapted with permission from ref 116. Copyright 2015, American Chemical Society. (g) Schematic of an encapsulated [Ni_3_(OH)_3_(H_2_O)_3_P_2_W_16_O_59_]^9–^ (Ni_3_P_2_W_16_) POM inside a NU-1000 pore (left) along with the photocatalytic H_2_ production values (right).

Adapted with permission from ref 111. Copyright 2020, Royal Society of Chemistry.

Adapted with permission from ref 113. Copyright 2013, Royal Society of Chemistry.

Adapted with permission from ref 114. Copyright 2022, Elsevier.

Adapted with permission from ref 117. Copyright 2021, Elsevier.

Studies exploring POMs as light-triggered catalysts can be dated back to early 1985, where they were initially used for H_2_ production *via* photochemical dehydrogenation of organic substrates.^93^ Over the past decades, POMs have been studied extensively^94,95^ and have triggered a lot of attention in homogeneous catalysis due to their structural tunability and stability toward oxidative decomposition in combination with rich acid–base properties.^96,97^ Importantly, due to a large structural variety of POM building blocks, it is further possible to incorporate various other metal ions into the POM structure by sandwiching them between main structural units (Figure 3a, left), giving rise to a versatile POM chemistry of Mn, Ni, Co, Ru, *etc.* that are of particular interest to redox catalysis.^98,99^ An important milestone in POM research has been reached in 2010 when, after initial works on a Ru-based system,^100–102^ Hill and colleagues reported the first all-inorganic, abundant-metal-based molecular catalyst that could perform visible light-driven water oxidation in the homogeneous phase.^103^ Their catalyst was a cobalt-based POM, featuring a nature-inspired {Co_4_O_4_} cubane-containing core, which self-assembled in water from simple building blocks.

Following this, many POM clusters have been designed and shown to act as promising multielectron redox catalysts toward water oxidation reaction^104^ and photocatalytic HER^105^ under homogeneous conditions. Given that the community also pursued the heterogenization of POMs to create superior acid–base catalysts for decades,^106^ it was natural to develop first molecular/inorganic photosystems involving POM clusters, which that are of relevance to this review.

#### 3.2.1 Surface Grafting

In an early 2008 study, Kato et al. employed a grafting approach to electrostatically anchor a novel Dawson-type dirhenium­(V)-oxido-bridged polyoxotungstate (P_2_W_17_Re)_2_ onto the TiO_2_ surface premodified with cationic moieties.^107^ The resulting photocatalyst composite was tested for its photocatalytic H_2_ production performance from water vapor without the use of any sacrificial agent under visible light illumination using two cutoff filters: 400 and 420 nm. In the first case, the authors observed a stable photocatalytic H_2_ production of 3.96 μmol h^–1^ g^–1^ over five catalytic runs with apparent quantum yields (AQY) values around 0.1%, outperforming a reference sample prepared by physically mixing the two components by an order of magnitude. However, in the second case (using 420 nm filter), the (P_2_W_17_Re)_2_/TiO_2_ resulted in a much lower H_2_ production rate, which confirmed the photosensitizing role of the wide bang gap titania and indicated the cocatalytic function of the Re-POM cluster. In a similar approach however relying on coordination bonding, Nandan et al. modified the TiO_2_ surface with 3-aminopropyltriethoxysilane (APTES) sites to provide anchor points suitable for the attachment of a Co-substituted Co_2_W_11_ Keggin cluster known for its activity toward light-driven water oxidation under homogeneous conditions.^108,109^ As a result of mixing the POM and APTES/TiO_2_ in the solution, the aqua ligand of the Co^2+^ center could be replaced by a stronger NH_2_ from APTES, resulting in a H_2_N: → Co^2+^ dative bond formation and self-assembly of the components on TiO_2_, which was further corroborated with scanning transmission electron microscopy (STEM) resolving individual POM clusters on the inorganic surface (Figure 3b). The resulting Co_2_W_11_/TiO_2_ composite showed a stable photocatalytic O_2_ evolution over the course of up to 15 h, whereas a benchmark Co_2_W_11_/[Ru­(bpy)_3_]^2+^ molecular dyad deactivated quickly likely related to the rapid degradation of the Ru-photosensitizer (Figure 3b). This work shows that heterogenization of molecular (co)­catalysts is a viable strategy to enhancing their long-term stability; it also demonstrates the advantage of using the POM family of molecular clusters benefiting from the possibility of their structural modification and incorporation of catalytically active sites. An excellent example for the latter point was reported by Liu et al., who heterogenized a diplatinum-modified Keggin POM^110^ to arrive at a Pt_2_W_11_/TiO_2_ hybrid photosystem (Figure 3c).^111^ This strategy allowed the authors to achieve H_2_ production values of ∼11 mmol h^–1^ g^–1^, almost three times the value obtained in benchmark Pt/TiO_2_ based on particulate Pt under similar conditions. A relatively stable photocatalytic performance over 10 cycles further suggested that atomic Pt incorporation into the POM cluster structure is a promising way to prevent Pt aggregation and sintering, which often results in gradual activity loss for photosystems based on traditional NP-based cocatalysts.

Apart from electrostatic- or coordination-bond-driven assemblies, strategies employing one-step *in situ* anchoring approaches have also been explored. In 2019, Zheng et al. used a hydrothermal approach to anchor a Ta_6_ POM onto CdZnS to create a photosystem capable of visible light absorption and catalysis. The resulting Ta_6_/CdZnS composite showed a uniform distribution of the clusters on the surface – as revealed by elemental mapping – and demonstrated outstanding HER performance reaching a H_2_ production rate of 43.05 mmol h^–1^ g^–1^ (AQY of 37%), reportedly the highest for tantalum-based POMs at the time including their homogeneous photosystems. Further optoelectronic characterizations revealed a direct charge extraction from the conduction band (CB) of the CdZnS to the lowest unoccupied molecular orbital (LUMO) of Ta_6_, where the HER reaction takes place.^112^

#### 3.2.2 Encapsulation

In an alternative approach toward heterogenization of POMs, in 2013, Zhang et al. prepared a tricomponent photocatalyst system sandwiching a PW_12_ POM between a CdS substrate and Au NPs (Figure 3d).^113^ The authors reported the synergistic effect between the components resulting in a 3-fold increase in the photocatalytic HER performance as compared to the bicomponent Au/CdS photosystem. This effect was attributed to the dual function of the phosphotungstate cluster, where it acted as a bridge between the CdS and Au NPs enhancing charge shuttling while also providing active H* adsorption sites. Expanding on this embedment strategy, Ding et al. developed a template-assisted synthetic protocol to self-assemble electrophilic POM clusters into the interstitial space between CdS and SiO_2_ forming a core–shell architecture (Figure 3e).^114^ The authors demonstrate that a number of sandwich POM compounds – along with a unique Fe_11_ ^115^ core – stabilized by lacunary {SbW_9_} fragments can be trapped inside a p-SiO_2_–NH_3_@CdS structure leading to improved and highly stable photocatalytic HER performance.

Another prominent but still underexplored encapsulation strategy involves the introduction of catalytic POM clusters inside the porous channels of photoactive microporous solids. The first study that incorporated a P_2_W_18_ cluster into the UiO-type metal–organic framework (MOF) built from visible light-absorbing [Ru­(bpy)_3_]^2+^-derived dicarboxylate ligands was reported only in 2015 (Figure 3f).^116^ The authors demonstrated a high loading of the clusters inside the framework and their homogeneous distribution. They further showcased an efficient quenching of the excited Ru-based metal-to-ligand charge transfer (^3^MLCT) emission by encapsulated P_2_W_18_, indicating facile electron transfer between the two components. The resulting P_2_W_18_/MOF photosystem was active in light-driven HER with TONs reaching 540, which is 13 times higher than that of the homogeneous control, indicating the cocatalytic role of the POM and high degree of stabilization achieved by its encapsulation. A more recent study loaded a NU-1000 MOF with two Ni-POMs, namely, Ni_3_PW_10_ and Ni_3_P_2_W_16_ via a wet impregnation approach (Figure 3g).^117^ The authors conclude a homogeneous POM distribution in the pores of the MOF based on the observed decrease in the surface area and the adsorption volume in correlation with the POM size. The Ni_3_P_2_W_16_/NU-1000 photosystem showed excellent photocatalytic HER performance reaching H_2_ generation rate of over 13,000 μmol h^–1^ g^–1^, which was 3–4 times higher against the Ni_3_PW_10_/NU-1000 analogue. This result highlights that the {WNi_3_O_4_} core is the likely catalytically active center of both the POMs; however, the final activity of the surface-anchored cluster is strongly defined by the choice of the stabilizing trilacunary unit with PW_9_ outperforming the larger P_2_W_15_. Although not conducted in this study, DFT calculations would have been valuable to reveal the details of this effect and to link the POM cocatalyst structure with its HER performance.

### 3.3 Secondary Building Units

Over the past 25 years, MOFs have developed into a multifunctional class of designer organic–inorganic hybrid materials. Relying on the concepts of coordination chemistry, hard–soft acid–base principle, and isoreticularity, more than 100,000 MOF structures have been accessed.^118^ Whereas the last subsection explored MOFs as porous supports suitable for the encapsulation of small clusters like POMs or thiometalates, this section will discuss another particular aspect of MOFs relevant to this review, namely, the idea of secondary building units (SBUs) as cocatalytic centers. SBUs are defined as inorganic metal-containing clusters or nodes that serve as the connecting points for organic linkers in MOFs, which by default makes them strongly relevant to the idea of catalytically active all-inorganic cocatalysts with molecule-like properties. The following sections will discuss several points related to discrete SBU-like clusters and MOF-derived and cage-like clusters and highlight framework heterogenization as a strategy to integrate such clusters into a solid-state semiconducting matrix.

#### 3.3.1 Discrete Clusters

Well-defined inorganic metal-oxo clusters comprising MOF SBUs represent a rich, yet underexplored, class of cluster cocatalysis. While most SBUs are typically associated with and are an integral part of a given MOF family, many archetypal SBUs can also exist as discrete molecular units independent of being stabilized within the extended framework. As this section showcases, numerous iconic MOF SBUs have been successfully isolated and characterized as discrete clusters.

MOF-5 is one of the most famous structures reported by Yaghi et al. back in 1999.^119^ It features the iconic tetrahedral Zn_4_ SBU linked by terephthalate ligands to form a 3D framework. From the structural point of view, this Zn_4_ unit is highly related to zinc oxocarboxylates – a class of multinuclear molecular clusters with the general formula Zn_4_O­(O_2_CR)_6_ that has been known for decades.^120–122^ Fueled by the interest in MOF exploration, a lot of fresh work has appeared where well-defined Zn-oxo clusters reminiscent of this Zn_4_ SBU have been designed and applied in various catalytic processes unravelling their reactivity and dynamics (Figure 4a).^123,124^ MIL-101 is another famous MOF especially known for its outstanding water stability.^125^ In contrast to the fully saturated tetrahedral Zn_4_ unit of the MOF-5, MIL-101 is built of trimeric M_3_ SBUs that possess open metal sites rendering it highly relevant in catalytic applications and redox processes. Discrete triangular bridged metal complexes have also been known in the community since a while, with several families of M_3_O­(O_2_CR)_6_ centered V, Cr, Fe, and Mn clusters individualized, characterized, and studied toward their electronic and magnetic properties.^126,127^ It has been also demonstrated by Peng et al. that it is possible to control the composition of the metals in such M_3_ clusters and produce predefined Fe/Cr, Fe/Co, Fe/Ni M_3_ units to be incorporated into a well-defined mixed-metal MIL-101 frameworks (Figure 4b).^128^ UiO-66 is another well-studied MOF known for its reach defect-chemistry.^129^ It contains a persistent Zr_6_ SBU that has been of interest to inorganic chemists for decades due to its ligand-shell tenability, the possibility to expand its cluster core, and catalytic activity (Figure 4c).^130–132^ In a similar way, driven by the desire to discover new building blocks to design MOFs or to understand MOF formation process, a lot of synthetic and fundamental research has been conducted on discrete Zr-oxo clusters and their analogs.^133^

**Figure 4:**
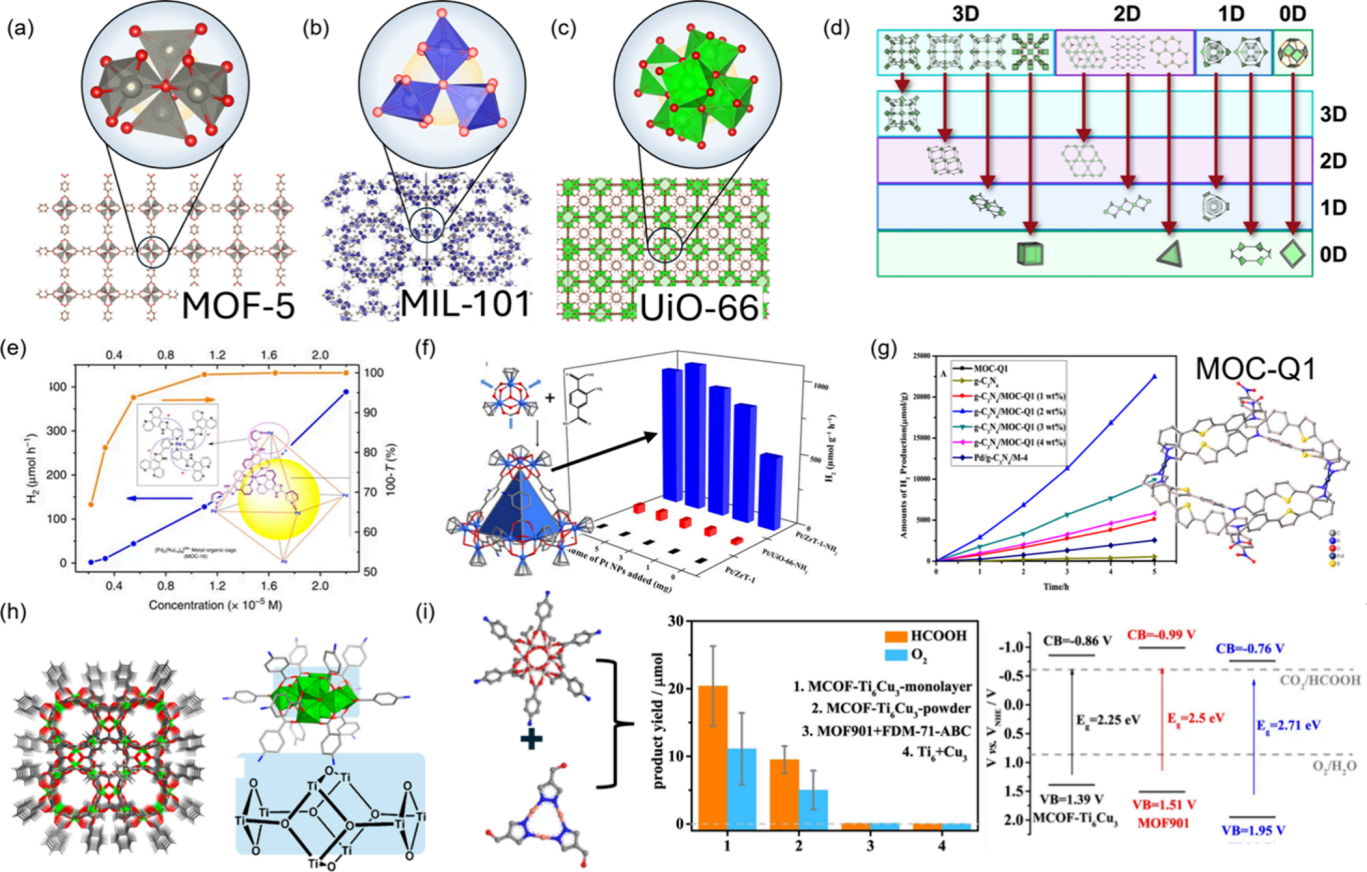
(a) MOF-5-Zn along with its Zn_4_O­(CO_2_)_6_ SBU. (b) MIL-101-Fe along with its Fe_3_O­(OH)_3_(CO_2_)_6_ SBU. (c) UiO-66-Zr MOF along with its Zr_6_O_4_(OH)_4_(CO_2_)_12_ SBU. (d) Clip-off chemistry approach allows isolation of discrete fragments and clusters from framework materials. (e) Photoabsorption completeness (orange) and HER rate (blue) dependence on the concentration of the [Pd_6_(RuL_3_)_8_]^28+^ MOC along with its cage structure. (f) Synthesis scheme of [(Cp_3_Zr_3_O­(OH)_3_)_4_L_6_]^4+^ (ZrT-1) MOP along with its photocatalytic HER rates compared with reference materials. (g) HER rates of [Pd_2_L_4_(BF_4_)_4_] (Q1) MOC heterogenized on C_3_N_4_ were shown along with the structure of Q1. (h) Cylindrical packing structure (left) and geometry of the discrete Ti_6_O_6_(L)_6_(OPr)_6_ (Ti_6_) cluster (right). Adapted with permission from ref 147. Copyright 2013, American Chemical Society. (i) Individual components of the Ti_6_Cu_3_ MOF integrating the Ti_6_-like SBUs (left), photocatalytic product yields from different sample mixtures (middle), and their respective energy levels (right).

Adapted with permission from ref 134. Copyright 2021, WILEY-VCH.

Adapted with permission from ref 138. Copyright 2016, Springer Nature.

Adapted with permission from ref 140. Copyright 2021, WILEY-VCH.

Adapted with permission from ref 145. Copyright 2021, American Chemical Society.

Adapted with permission from ref 148. Copyright 2022, Springer Nature.

These and many other discrete SBU-like clusters possess the same well-defined metal-oxo core structures and coordinatively unsaturated metal sites that often underpin catalytic activity within their parent MOFs. Their existence as soluble, characterizable molecules offers a direct pathway to utilize them as heterogenizable cocatalysts on semiconductor supports – bypassing the need for the full MOF architecture when its porosity or additional complexity is unnecessary for the target photocatalytic function.

#### 3.3.2 SBU Cleavage

While in the examples above, metal-oxo clusters have mostly been accessed independently for their relevance to MOFs, it is also possible to use existing MOF structures and individualize their unique, otherwise inaccessible SBUs. The concept of clip-off chemistry, pioneered by Maspoch et al.,^134^ provides a powerful tool for accessing discrete novel SBU-like clusters (Figure 4d). This approach involves the deliberate disassembly of coordination networks like MOFs^135^ and covalent-organic frameworks (COFs)^136^ through selective bond cleavage to isolate well-defined, metal–organic cages (MOCs) and metal–organic polyhedra (MOPs). From the perspective of cluster design and use as molecular cocatalysts, MOCs and MOPs represent a fascinating subclass of compounds featuring defined inorganic cores, tunable organic periphery, and unique porous microenvironments for substrate binding or reaction modulation.

Despite the field of MOCs/MOPs having gained momentum for decades^137^ and their potential having been explored in various fields of homogeneous catalysis (e.g., for organic transformations, H_2_ production, and CO_2_ reduction, Figure 4e,f),^138–140^ a critical gap in their heterogenization persists. While MOPs have been recently shown to be stabilized inside colloidal gel networks or porous matrices,^141,142^ there are no reported examples of MOPs deliberately integrated within semiconductor supports for application as cocatalysts in light-driven processes like HER or CO_2_RR. This absence is striking compared to the established field of POM or thiometalate anchoring discussed in Sections 3.1 and 3.2; however, it can be explained by the relative youth of the field, which still mainly focuses on the MOP design and assembly and prefers homogeneous applications over their integration into heterogeneous assemblies. MOCs, on the other hand, have been explored more intensely driven by their potential in homogeneous light-driven redox catalysis and relative ease of fabrication via self-assembly routes.^143^ To this date, Liu et al. provided several strong examples of MOC heterogenization on inorganic (TiO_2_, C_3_N_4_)^144,145^ and organic (COF)^146^ semiconductors to be used cocatalysts for HER. In all these cases, Pd-based M_2_L_4_ or M_3_L_2_ cages featuring catalytically active Pd^2+^ sites have been used as showcased in Figure 4g. Based on the results, Pd-MOCs show excellent ability to separate charges and cocatalyze the desired reaction, while maintaining their structural integrity. It was also demonstrated that Pd-MOCs outperform commonly used nanoparticulate Pd- or Pt-based cocatalysts. Despite these promising results, however, little has been done toward rational design and control of the structure and composition of the heterogenized MOCs, thus leaving a lot of room for follow-up work.

#### 3.3.3 Framework Heterogenization

While previous sections mostly talked about clusters that are related to common MOF SBUs, the current literature is rich with other structurally unique cluster compounds that should not be omitted from the scope of this review. An excellent example was provided by Chun and Hong in 2013, who managed to crystallize a Ti_6_ oxo-cluster (Figure 4h) that adopts a hexagonal column geometry stabilized by 4-aminobenzoic acid ligands.^147^ Almost a decade later, this unique cluster has been turned into an SBU to construct a heterometallic Ti–Cu MOF featuring oxidative and reductive fragments (Figure 4i). This co-operation of (co)­catalytic centers and their close proximity inside the framework enabled excellent photocatalytic performance toward HCOOH generation from CO_2_ and H_2_O.^148^ Extensive DFT modeling further revealed Ti_6_ SBU to act as a sink for photogenerated holes and as an active site for water oxidation.

From the point of view of turning a molecular cluster into a surface-supported cocatalyst, this strategy can be seen as an example of “framework heterogenization”, in which the cluster turns into an important building unit of the photoactive solid-state matrix. This idea is largely underexplored mostly due to the complex set of geometric and structural requirements an SBU should possess to be suitable for MOF construction; however, a similar concept has been long known in the field of POM-based MOFs – sometimes abbreviated as POMOFs^149^ – who are a small subset of MOFs in which SBUs are constituted by individual POM clusters. Importantly, POMOFs are very different from cases where POM clusters are simply embedded inside the MOF pores – such as those discussed in section 3.2.2. The synthesis of such a POMOF typically starts with a presynthesized POM of a given structure and composition, which is then assembled with the properly chosen ligands to yield a 3D framework. Formation of such a POMOF can thus be seen as a way of POM heterogenization, so in order to take advantage of the POM SBUs as well-defined cocatalysts incorporated into a MOF, several considerations need to be taken into account. Since POMs are not well-known for their photoactivity in the absence of a suitable molecular photosensitizer,^104,109,150,151^ the most straightforward way to ensure the framework photoresponsiveness has been by using ligands capable of UV or visible light absorption followed by a ligand-to-metal charge transfer (LMCT) given the ability of POMs to accommodate excess electrons.^152^ Second, the POM cluster must be catalytically relevant. While a large number of POMOFs are known in the community, so far, most common SBU is the {ε-Zn_4_PMo_12_} unit.^153^ While basic Anderson, Lindqvist, Keggin-based POMs are known to facilitate acid–base catalytic reactions, it is common that transition-metal substitution or incorporation is necessary to turn a POM cluster into the one catalytically active toward multielectron redox processes such as HER and OER.^154–156^ More work is expected to be done in the direction of framework heterogenization of catalytically relevant POM and other oxo clusters in the future.

### 3.4 Metal Nanoclusters (NCs)

Metal NCs are typically composed of a metallic core with a defined number of atoms surrounded by covalently bound organic ligands. The metal core generally plays a major role in enabling the catalytic behavior and defines optoelectronic properties of the NC, whereas the enveloping ligands protect and regulate its chemical interaction with different surroundings (Figure 5a). From their first discovery in the late 1970s,^157,158^ NCs have been studied for over four decades, with major challenges mostly linked to their synthetic development, isolation, and chemical stability. Due to their well-defined electronic and geometric structure, which enables understanding of structure–activity relationships, these NCs have been thoroughly explored as model systems in the field of heterogeneous catalysis^158,159^ and photocatalysis.^160,161^

**Figure 5:**
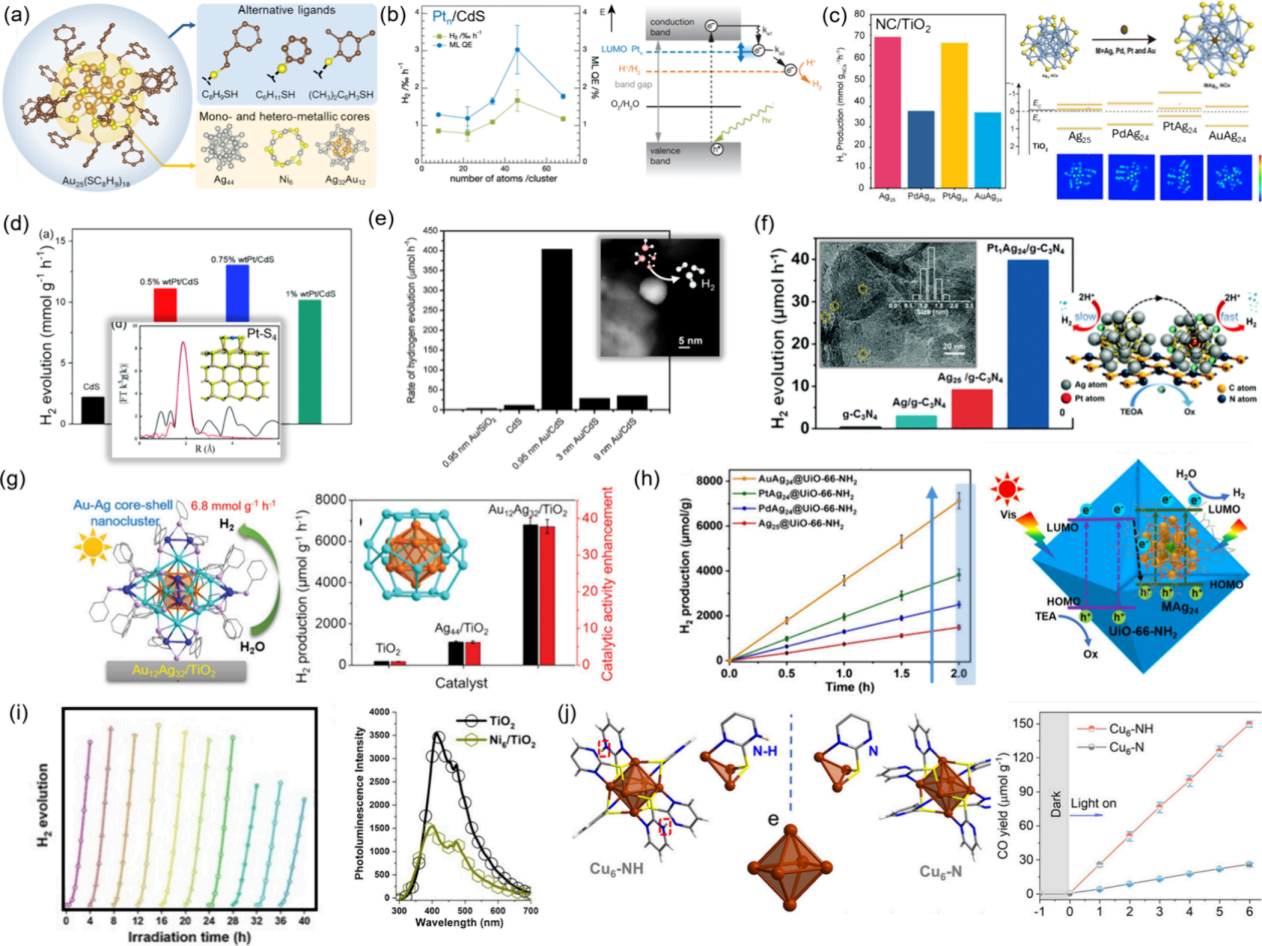
(a) Schematic representation of a Au_25_(SG)_18_ NCs with a metallic-like core (left), the commonly used ligands (top right) and examples of other reported NC cores (right bottom). (b) Photocatalytic performance of Pt_n_/CdS nanorods as a function of Pt_*n*_ NC size (left) and the proposed reaction pathway for H_2_ production under illumination (right). Adapted with permission from ref 166. Copyright 2013, American Chemical Society. (c) Photocatalytic HER rates (left) and a schematic showing the structure of pristine and heterometallic MAg_24_ NCs (right top) along with their corresponding theoretical energy level positions and charge density plots (right bottom). (d) Photocatalytic HER rates were obtained with different Pt_5_ NC loadings. Inset: EXAFS R-space fitting curves with a structural representation of the Pt–S_4_ link. (e) HER rates for Au/CdS composites comparing Au_11_ NC with Au NPs of varying size. Inset: STEM image showing 9 nm Au loaded on CdS. (f) HER activities comparing Pt-incorporated Pt_1_Ag_24_ vs pristine Ag_25_ heterogenized on C_3_N_4_ (left) along with proposed enhancement mechanism (right). Inset: TEM analysis of the NC size distribution. (g) Schematic showing the core–shell Au_12_Ag_32_ NC structure (left) and its photocatalytic performance compared to the pristine Ag_44_ cluster (right). Inset: Au_12_Ag_32_ polyhedral core. (h) Time-dependent photocatalytic HER profiles of various noble-metal heterometallic NCs loaded onto the UiO-66-NH_2_ MOF (left) and the proposed Z-scheme photocatalytic mechanism (right). (i) HER profiles showing stable performance of Ni_6_ NC for 10 photocatalytic cycles (left) and the quenching of TiO_2_ PL emission spectra in the presence of Ni_6_. (j) Schematic representation of Cu_6_–N and Cu_6_–NH NC structures (left) and their respective CO_2_ photoreduction performance (right).

Adapted with permission from ref 167. Copyright 2023, WILEY-VCH.

Adapted with permission from ref 168. Copyright 2022, Royal Society of Chemistry.

Adapted with permission from ref 172. Copyright 2012, Elsevier.

Adapted with permission from ref 174.

Adapted with permission from ref 175. Copyright 2023, WILEY-VCH.

Adapted with permission from ref 176. Copyright 2024, WILEY-VCH.

Adapted with permission from ref 179. Copyright 2021, Elsevier.

Adapted with permission from ref 182. Copyright 2023, WILEY-VCH.

It is important to note that due to the molecular nature of metal NCs featuring discrete energy levels, small changes in the number of atoms can lead to a pronounced change in their band gaps and optoelectronic characteristics. Exploring this aspect has led to extensive research on the light-absorbing properties of NCs and the prospects of their heterogenization. In a typical architecture, the NCs are anchored onto catalytically active metal oxide or sulfide surfaces where the NCs play the role of the light absorber forming photogenerated electrons, which are then transferred to the support and carry out the reaction.^162^ While this photoresponsiveness of NCs is somewhat unique, more recently, the interest has shifted to investigating NCs as efficient cocatalytic systems when coupled with light-absorbing semiconductors.^163^ Within this realm, several studies have shown that reactivity mechanisms can widely differ based on the type of semiconductor (SC) that they are anchored on. For instance, since the Fermi level and thereby the LUMO of most NCs typically lie higher than that of most wide band gap n-type semiconductors, these NCs often tend to form a staggered type-II heterojunction.^161^ This results in the electron transfer barrier from the SC to the NCs, thus limiting their use as a cocatalyst. In contrast, p-type semiconductors readily form junctions that facilitate the electron transfer from the CB of the SC to the LUMO of the NC, thus making it available for further redox reaction.

Due to the scope of this review on cluster heterogenization for the benefit of the well-defined catalytic active sites, here we will only focus on the function of these NCs to act as cocatalysts (and not as light absorbers), along with summarizing the role of organic ligands reflected in their overall photocatalytic performance for HER. The following subsections will highlight relevant literature and discuss it based on the composition of the NC metal core covering homometallic noble-metal, heterometallic noble-metal, and non-noble-metal NCs.

#### 3.4.1 Homometallic Noble-Metal Nanoclusters

Currently, several homometallic noble-metal nanoclusters of Au, Ag, Pt, and Ir have been successfully synthesized. While all these noble metals are generally known for their catalytic excellence, from the point of view of the application in photocatalytic HER and considerations of the optimal H* adsorption energy,^164,165^ Pt stands out as the most active HER cocatalyst. Motivated by this, Jäckel et al.^166^ decorated CdS with Pt-NC via laser ablation and successfully applied the resulting photocatalytic system to light-driven HER. The proposed deposition technique allowed them to probe the effect of different cluster coverages on the overall performance. In a follow up study,^166^ the authors further elucidated on the effect of cluster size varying from Pt_8_ to Pt_68_, revealing an initial increase in HER activity with increasing cluster size and showing that optimal performance stem from a Pt_46_ NC (Figure 5b). This trend was rationalized based on the variability of the LUMO position of the NC depending on its number of atoms. Considering the major thermodynamic driving forces in the Pt_*x*_/CdS photosystem, the LUMO of the NC must lie between the CB of CdS and the hydrogen reduction potential. The optimal Pt_46_ not only fulfills this basic requirement but it also allows a compromise between the efficient charge extraction from CdS while retaining sufficient reduction potential to carry out the HER reaction. A similar conclusion was reached when studying heteroatom-doped Ag_25_ NCs – discussed in detail in the next subsection – where their doping with Pt, Pd, or Au resulted in an upward (positive) shift of the LUMO level (Figure 5c).^167^ While precise control of the optoelectronic properties of NCs can be used to tune charge-transfer dynamics at the heterojunction interface, the use of well-defined Pt_5_ NCs deposited on CdS recently allowed pinpointing of the active HER sites and rationalizing high visible light-driven HER of the NC/CdS photosystem.^168^ Extended X-ray absorption fine structure (EXAFS) spectra revealed a partial positive charge on the Pt centers in the composite, helping to identify Pt–S_4_ species (Figure 5d) as most likely active charge transfer channels. Furthermore, first-principles calculations of the Δ*G*_H*_ values for adsorption on isolated Pt atoms were shown to be much closer to the optimal zero value as compared to other possible H-adsorption sites.

In contrast to Pt NCs that are relatively underexplored in their applications toward light-driven solar fuel production, thiol-protected Au NCs have been investigated more thoroughly given their high accessibility and spread.^39,169,170^ Purely from a cocatalytic standpoint, the Au–S bonds have been shown to improve the photoelectrocatalytic performance of MoS_2_ sheets by reducing the onset HER potential.^171^ Building on this prospect, small Au NCs were for the first time employed as HER cocatalysts by anchoring subnm Au_11_ NCs onto semiconducting CdS.^172^ Not only did the authors observe a 35-fold HER boost over bare CdS enabled by the heterogenized NCs, they also showcased that Au_11_ outperformed conventional particulate cocatalysts including Au, Pt, and Pd NPs in the size range between 2 and 4 nm (Figure 5e). While the study did not provide exact underlying reason for observing such a drastic improvement in the photocatalytic performance, high activity of Au_11_ can be well related to their higher surface-to-bulk ratio and the proportion of catalytic Au centers at the cocatalyst surface. This conclusion was verified by Negishi et al., who for the first time investigated larger Au_25_ NCs deposited onto BaLa_4_Ti_4_O_15_ via a facile wet impregnation method and demonstrated their superior cocatalytic HER performance compared to Au NPs.^173^

#### 3.4.2 Heterometallic Noble–Metal Nanoclusters

The optimal H* adsorption energy on the Pt atom makes it an attractive active site to be incorporated into a heterometallic NC. In 2017, Yang et al. reported the first 1.5 nm large Pt_1_Ag_24_ NC anchored onto a visible light-absorbing C_3_N_4_ substrate.^174^ This heteroatom NC resulted in a photocatalytic improvement by a factor of 4 as compared to the pure Ag_25_ NC (Figure 5f), which could be attributed to the catalytic role of Pt sites in improving the sluggish kinetics and poor electron trapping ability inherent to Ag-based catalytic systems. EXAFS was instrumental in this work to unravel the Pt–Ag bonding, thus excluding cluster decomposition or Pt migration upon the photocatalytic reaction and further emphasizing the positive effect of Pt incorporation. A similar concept based on compositional tunability of NCs was used to design a templated galvanic exchange strategy and replace 12 Ag atoms of Ag_44_ NC core with Au (Figure 5g).^175^ The resulting negatively charged Au_12_Ag_32_ was electrostatically anchored onto the TiO_2_ surface – which bore a positive charge due to oxygen deficiency – resulting in a photocatalytic H_2_ production rate of 6810 μmol h^–1^ g^–1^, 6-fold higher than that of the pristine Ag_44_/TiO_2_ system. Interestingly, the authors ascribed this improvement not to the formation of efficient catalytic sites due to Ag-to-Au replacement but to the improved charge extraction due to better-aligned energy levels at the Au_12_Ag_32_/TiO_2_ interface according to the proposed Z-scheme heterojunction uncovered using PL spectroscopy. This work one more time emphasizes the dual role of metallic NCs as light collectors and cocatalysts, suggesting that their exploration needs to consider both the effects and their mutual co-operation.

One insightful contribution to the topic of NC modulation was provided by Wang et al., who attempted to dope a unique core–shell Ag_25_ NC with single Au, Pt, and Pd atoms, however only replacing the position of the central Ag (Figure 5c).^167^ The resulting Au_1_Ag_24_, Pt_1_Ag_24_, and Pd_1_Ag_24_ were deposited onto TiO_2_ and benchmarked toward their photocatalytic HER performance. In contrast to previous examples, Au and Pd atom doping resulted in a strong decrease in the photocatalytic performance, while Pt doping did not seem to have much effect. This result could be related to the inaccessibility of the central metal site for catalysis, whereas the main negative effect of single Au and Pd incorporation likely stemmed from the energy level mismatch upon doping, which was confirmed based on ultraviolet photoelectron spectroscopy (UPS) and XPS studies along with DFT modeling. A similar study was conducted by Jiang et al., who looked into the effect of noble-metal-doped Ag_25_ clusters encapsulated inside UiO-66 MOF pores (Figure 5h) – used here to ensure higher cluster loading without the fear of agglomeration.^176^ Contrary to the previous case, the authors observed a strong improvement in the photocatalytic performance upon cluster doping in the following order: AuAg_24_ > PtAg_24_ > PdAg_24_ > Ag_25_. PL and XPS studies pointed to an efficient charge extraction via a Z-scheme-based charge transfer mechanism. Moreover, electron densities measured for each sample showed that the central atom modulates (decreases) the electron density on the Ag atoms, which is reflected in the photocatalytic performance. While the previous studies compared the effect of doping in the same Ag_25_ NC, Zhu et al. explored the effect of Pt-atom substitution into structurally defective Ag_*x*_ NCs of various sizes (*x* = 9, 11, 13, 14) anchored onto C_3_N_4_.^177^ The highest activity was observed for the Pt_1_Ag_11_/C_3_N_4_ case and attributed to the formation of a Z-scheme heterojunction in which the Pt_1_Ag_11_ cluster shows the lowest Gibbs free H* adsorption energy. DFT calculations further suggested that the observed charge extraction/transfer could be facilitated due to the Ag–N bond formation and π–π interactions. While the different catalytic trends reported in these three studies can be explained by the difference in the chosen supports (oxide vs MOF vs nitride), they showcase the high level of structural insights and control over charge extraction/release that can be gained by employing structurally defined NCs as cocatalysts.

#### 3.4.3 Non-noble-Metal Nanoclusters

Non-noble-metal nanoclusters have been of interest to the community since as early as 2013 when the use of Ni_6_(SR)_12_-like complexes forming a crown-like cycle framework toward photocatalytic HER was demonstrated under homogeneous conditions.^178^ Only recently, Chen et al. reported heterogenization of a similar Ni_6_ NC that was anchored onto TiO_2_ via a solvent evaporation method. The resulting Ni_6_/TiO_2_ composite showed excellent light-driven H_2_ production of ∼5600 μmol h^–1^ g^–1^ with over 10 cycles of stable activity whereas a combination of PL and transition absorption spectroscopy (TAS) studies confirmed a charge transfer pathway from the TiO_2_ CB to the Ni_6_ upon excitation (Figure 5i). DFT modeling provided a deeper picture on the underlying photocatalytic mechanism, revealing that the phenyl groups of the Ni_6_ core protecting ligands lie parallel to the TiO_2_ surface, thereby allowing for an overlap between their π orbitals and the p orbital of the surface oxo ligands of the TiO_2_. This suggests that the Ni_6_/TiO_2_ forms a pseudo-Z-scheme via the phenyl groups acting as a bridge between the Ni-NC and TiO_2_.^179^

Cu-based compounds have also gained attraction for their potential as earth-abundant cocatalysts toward photocatalytic solar fuel generation processes.^180,181^ Recently, Wang et al. reported successful synthesis of Cu_6_ NCs featuring a distorted Cu_6_ octahedron stabilized by six 2-mercaptopyrimidine ligands that alternate in orientation (Figure 5j, left).^182^ The authors managed to isolate two Cu_6_ compounds that differed in protonation of the N atom on the pyrimidine ring resulting in isostructural Cu_6_–N and Cu_6_–NH clusters, which – despite their structural similarity – showed drastically different abilities in catalyzing light-driven CO_2_RR in the gas phase (Figure 5j, right). Based on diffuse reflectance infrared Fourier transform spectroscopy (DRIFTS) and DFT evidence, this difference was explained by the ability of protonated pyrimidine N atoms in Cu_6_–NH to provide a local proton effectively facilitating proton-coupled electron transfer involved in CO_2_ activation, which again highlights the importance of atomic configuration in enabling high-performing catalytic properties of a given cluster. While this initial report demonstrated that these unique Cu_6_ clusters act as self-sensitizing semiconductors, it is intriguing to explore their anchoring onto light-absorbing substrates, alleviating challenges such as cluster agglomeration and stability.

## 4 Conclusions and Outlook

This review has provided a comprehensive look into the burgeoning field of atomically precise, molecular clusters with all-inorganic cores – thiometalates, MOF-derived SBUs, POMs, and metallic NCs – serving as cocatalysts in light-driven catalytic reactions. The showcased examples undeniably document their beneficial role in enhancing the photocatalytic performance. More significantly, they highlight the unique possibility offered by their structural and compositional control: the rational tuning of optoelectronic properties (e.g., band alignment, light absorption) and catalytic characteristics (e.g., active site density, binding energy) to optimize both charge extraction from the semiconductor and charge utilization in the target redox process (e.g., HER, OER, CO_2_RR). Our critical assessment of the existing literature reveals several knowledge gaps and formulates recommendations for future directions in this exciting research field.

First, the potential of the structural tunability of these clusters needs to be capitalized on. For this, studies involving clusters explicitly designed with mechanistic questions in mind need to be given priority. As such, clusters with modified ligands (their type or number) and metal centers (nuclearity and core geometry) are of high interest. Our further recommendation is to conduct these fundamental studies based on well-understood and controlled semiconducting supports (e.g., TiO_2_, Al:SrTiO_3_, well-characterized graphic C_3_N_4_) to avoid a complex overlap of different effects arising from the support and the cocatalyst parts of the equation. Ultimately, faceted semiconducting nanocrystals with well-defined surface structures and chemistries are the most preferred candidates as they allow for complete interfacial control and rational construction of DFT models.^183–185^

Second, the use of well-defined cocatalysts shall allow us to understand their reactivity and dynamics on an unprecedented level; however, advanced (surface-sensitive) *in situ/operando* characterization needs to be developed and employed given the low expected content of the cocatalytic component in the final photosystem. Prominent examples of high resolution electron microscopy,^109,186^ scanning tunneling microscopy,^187,188^ atomic force microscopy,^189^ and synchrotron-based X-ray absorption studies^190^ can be highlighted, but advanced spectroscopic techniques like surface-sensitive infrared reflection–absorption spectroscopy (IRRAS) or time-resolved characterization tools are yet to be explored. Additionally, advance periodic DFT methods need to be applied to simulate cluster/support interfaces similar to the work done on supported catalysts.^191–193^

Third, we believe that in order to harness the unique advantages of molecular cluster cocatalysts toward facilitating photocatalyst design and discovery, future research must adopt a more critical and rigorous approach toward performance evaluation. As such, the community needs to develop and implement standardized protocols to assess cocatalyst stability and activity under operational conditions (light, potential, solvent, reactants). While this is a part of the general concern in the field of photocatalysis,^194–199^ the use of well-defined cluster cocatalysts uniquely allows reporting TOF and TON metrics accepted in the field of homogeneous photocatalysis. Further, it makes it possible and advantageous to benchmark the heterogenized cocatalyst photosystem with its homogeneous counterpart, which brings useful mechanistic insights as has already been demonstrated in some rare cases.^84,109,200^ As of now, unfortunately, the majority of the reported works still stick to highly subjective absolute or mass-normalized activity rates (in μmol h^–1^) and do not reveal or measure photon fluxes (Table S1), which makes it virtually impossible to compare activities across different works and research laboratories.

Fourth, as this review showcases, the community should more proactively explore the vast chemical space of underexplored cluster types to achieve superior functionalities. Thiometalates have rich chemical and structural variety that stretches far beyond the prototypical [Mo_3_S_13_]^2–^ compound. The possibility to replace terminal disulfides with organic ligands shall also be exploited for targeted heterogenization of these clusters into semiconducting surfaces as well as their integration into the backbone of the metal–organic and covalent-organic frameworks. Heterometallic and sandwich POMs have the possibility to incorporate redox-active metals and thus provide an ideal platform for turning often inactive POM structures into active cocatalytic clusters. For both POM and thiometalate heterogenization, synthetic protocols for irreversible anchoring and studies exploring leaching and cluster transformation are highly required. MOF-inspired or MOF-derived SBU-like clusters, MOPs, and MOCs provide another broad range of molecular cocatalysts that can be isolated, characterized, and heterogenized enabling well-defined catalytic centers and the possibility for purposeful design. The field of metal nanoclusters as light-driven cocatalysts still lacks systematic studies exploring the effect of core nuclearity on their activity to distinguish electronic vs catalytic effect of the cluster structure. Additionally, there seems to be a lack of understanding of the role and necessity of the ligands after heterogenization took place. Finally, other all-inorganic clusters such as chalcogenide T4 clusters^201,202^ – [Cd_3_In_17_Q_31_Cl_4_]^9–^ and [Cd_3_In_17_Se_31_]^5–^ – or a series of doped [Mn_4_Ga_14_Sn_2_S_35_]^12–^ reported back in 2010 have not, to the best of our knowledge, been explored toward their cocatalytic function.^203,204^

## Supplementary Material



## Notes

Table S1: summary table of various classes of cocatalysts on photoactive supports along with the reaction conditions and typical photocatalytic HER performance metrics reported (PDF)

## Vocabulary

Cocatalyst: A secondary material deposited on a photocatalyst semiconductor surface that accelerates charge separation by extracting photoexcited charge carriers and lowers activation barriers by providing active sites for desired chemical transformations.
Atomically precise clusters: Well-defined, molecular inorganic clusters with experimentally resolved three-dimensional structures at the atomic level, typically composed of 2–100 metal atoms, that bridge the size and structural gap between single-atom catalysts and nanoparticulate or quantum dot materials while maintaining compositional and structural uniformity.
Surface anchoring (or heterogenization): The covalent or electrostatic immobilization of molecular or cluster cocatalysts onto the surface of a solid-state semiconductor photocatalyst support, creating a hybrid system that combines the structural precision of homogeneous catalysts with the robustness and recyclability of heterogeneous systems.
Turnover frequency (TOF) and turnover number (TON): TOF is the number of catalytic cycles completed by a single active site per unit time (typically s^–1^); TON is the total number of substrate molecules converted per active site over the entire catalytic lifetime. Both metrics enable direct comparison of intrinsic catalytic performance between heterogenized cluster cocatalysts and their homogeneous molecular counterparts.
Structure–activity relationship: The quantitative or qualitative correlation between the atomic or molecular structure of a catalyst (composition, geometry, and coordination environment) and its catalytic performance (activity, selectivity, and turnover frequency), enabling rational design of improved catalysts.

## References

[1] Hultgren A., Carleton T., Delgado M., Gergel D. R., Greenstone M., Houser T., Hsiang S., Jina A., Kopp R. E., Malevich S. B., McCusker K. E., Mayer T., Nath I., Rising J., Rode A., Yuan J. (2025). Impacts of Climate
Change on Global Agriculture Accounting for Adaptation. Nature.

[2] Lèbre É., Stringer M., Svobodova K., Owen J. R., Kemp D., Côte C., Arratia-Solar A., Valenta R. K. (2020). The Social and Environmental
Complexities of Extracting Energy Transition Metals. Nat. Commun..

[3] Tay N. E. S., Lehnherr D., Rovis T. (2022). Photons or Electrons?
A Critical
Comparison of Electrochemistry and Photoredox Catalysis for Organic
Synthesis. Chem. Rev..

[4] Gunawan D., Zhang J., Li Q., Toe C. Y., Scott J., Antonietti M., Guo J., Amal R. (2024). Materials Advances
in Photocatalytic Solar Hydrogen Production: Integrating Systems and
Economics for a Sustainable Future. Adv. Mater..

[5] Uekert T., Pichler C. M., Schubert T., Reisner E. (2021). Solar-Driven Reforming
of Solid Waste for a Sustainable Future. Nat.
Sustain..

[6] Andrei V., Wang Q., Uekert T., Bhattacharjee S., Reisner E. (2022). Solar Panel Technologies for Light-to-Chemical Conversion. Acc. Chem. Res..

[7] Takata T., Jiang J., Sakata Y., Nakabayashi M., Shibata N., Nandal V., Seki K., Hisatomi T., Domen K. (2020). Photocatalytic Water Splitting with
a Quantum Efficiency of Almost
Unity. Nature.

[8] Nishiyama H., Yamada T., Nakabayashi M., Maehara Y., Yamaguchi M., Kuromiya Y., Nagatsuma Y., Tokudome H., Akiyama S., Watanabe T., Narushima R., Okunaka S., Shibata N., Takata T., Hisatomi T., Domen K. (2021). Photocatalytic Solar
Hydrogen Production from Water on a 100-M2 Scale. Nature.

[9] Low J., Zhang C., Ma J., Murzin D. Y., Xiong Y. (2023). Heterogeneous
Photocatalysis: What Is Being Overlooked?. Trends
Chem..

[10] Mendhe A., Panda H. S. (2023). Machine Learning-Assisted Electrode
Material Fabrication
and Electrochemical Efficiency Prediction and Validation of PANI-Ni/Co
Hydroxide Nanocomposites. ACS Sustain. Chem.
Eng..

[11] Yang K., Li Y., Zhang J. (2023). High-Throughput Screening
of Hybrid Quaternary Halide
Perovskites for Optoelectronics. J. Mater. Chem.
A.

[12] De
Vos J. S., Ravichandran S., Borgmans S., Vanduyfhuys L., Van Der Voort P., Rogge S. M. J., Van Speybroeck V. (2024). High-Throughput
Screening of Covalent Organic Frameworks for Carbon Capture Using
Machine Learning. Chem. Mater..

[13] Alghofaili Y. A., Alghadeer M., Alsaui A. A., Alqahtani S. M., Alharbi F. H. (2023). Accelerating Materials Discovery through Machine Learning:
Predicting Crystallographic Symmetry Groups. J. Phys. Chem. C.

[14] Yang J., Wang D., Han H., Li C. (2013). Roles of Cocatalysts
in Photocatalysis and Photoelectrocatalysis. Acc. Chem. Res..

[15] Li X., Yu J., Jaroniec M., Chen X. (2019). Cocatalysts for Selective Photoreduction
of CO2 into Solar Fuels. Chem. Rev..

[16] Wang Q., Hisatomi T., Jia Q., Tokudome H., Zhong M., Wang C., Pan Z., Takata T., Nakabayashi M., Shibata N., Li Y., Sharp I. D., Kudo A., Yamada T., Domen K. (2016). Scalable Water Splitting on Particulate
Photocatalyst Sheets with a Solar-to-Hydrogen Energy Conversion Efficiency
Exceeding 1%. Nat. Mater..

[17] Zhang W., Luo J., Tang H., Wang S., Li W., Zhang J., Zhou Y. (2024). Co-Doped RuO2
Nanoparticles with Enhanced Catalytic Activity and
Stability for the Oxygen Evolution Reaction. Dalton Trans..

[18] Haselmann G. M., Eder D. (2017). Early-Stage Deactivation
of Platinum-Loaded TiO2 Using In Situ Photodeposition
during Photocatalytic Hydrogen Evolution. ACS
Catal..

[19] Nørskov J. K., Bligaard T., Logadottir A., Kitchin J. R., Chen J. G., Pandelov S., Stimming U. (2005). Trends in
the Exchange Current for
Hydrogen Evolution. J. Electrochem. Soc..

[20] Maeda K., Teramura K., Lu D., Saito N., Inoue Y., Domen K. (2006). Noble-Metal/Cr2O3 Core/Shell Nanoparticles as a Cocatalyst for Photocatalytic
Overall Water Splitting. Angew. Chem., Int.
Ed..

[21] Schubert J. S., Doloszeski E., Ayala P., Myakala S. N., Rath J., Fickl B., Giesriegl A., Apaydin D. H., Bayer B. C., Kashiwaya S., Cherevan A., Eder D. (2024). Nature of the Active
Ni State for Photocatalytic Hydrogen Generation. Adv. Mater. Interfaces.

[22] Kanan M. W., Nocera D. G. (2008). In Situ Formation of an Oxygen-Evolving Catalyst in
Neutral Water Containing Phosphate and Co2+. Science.

[23] Steinmiller E. M. P., Choi K.-S. (2009). Photochemical Deposition of Cobalt-Based Oxygen Evolving
Catalyst on a Semiconductor Photoanode for Solar Oxygen Production. Proc. Natl. Acad. Sci. U. S. A..

[24] Garstenauer D., Nagaraju Myakala S., Ayala P., Rabl-Wolff H., Zobač O., Jirsa F., Eder D., Cherevan A., Richter K. W. (2025). Engineering
Active Intermetallic Pt–Zn Sites
via Vapour–Solid Synthesis for Photocatalytic Hydrogen Production. Sustain. Energy Fuels.

[25] Chinnabathini V. C., Dingenen F., Borah R., Abbas I., van der
Tol J., Zarkua Z., D'Acapito F., Nguyen T. H. T., Lievens P., Grandjean D., Verbruggen S. W., Janssens E. (2023). Gas Phase Deposition
of Well-Defined Bimetallic Gold-Silver Clusters for Photocatalytic
Applications. Nanoscale.

[26] Jin R., Li G., Sharma S., Li Y., Du X. (2021). Toward Active-Site
Tailoring in Heterogeneous Catalysis by Atomically Precise Metal Nanoclusters
with Crystallographic Structures. Chem. Rev..

[27] Yazaki D., Kawawaki T., Hirayama D., Kawachi M., Kato K., Oguchi S., Yamaguchi Y., Kikkawa S., Ueki Y., Hossain S., Osborn D. J., Ozaki F., Tanaka S., Yoshinobu J., Metha G. F., Yamazoe S., Kudo A., Yamakata A., Negishi Y. (2023). Carbon Nitride Loaded with an Ultrafine,
Monodisperse, Metallic Platinum-Cluster Cocatalyst for the Photocatalytic
Hydrogen-Evolution Reaction. Small.

[28] Rajapaksha R., Samanta P., Quadrelli E. A., Canivet J. (2023). Heterogenization of
Molecular Catalysts within Porous Solids: The Case of Ni-Catalyzed
Ethylene Oligomerization from Zeolites to Metal–Organic Frameworks. Chem. Soc. Rev..

[29] Mak C. H., Han X., Du M., Kai J.-J., Tsang K. F., Jia G., Cheng K.-C., Shen H.-H., Hsu H.-Y. (2021). Heterogenization
of Homogeneous Photocatalysts Utilizing Synthetic and Natural Support
Materials. J. Mater. Chem. A.

[30] Serna P., Gates B. C. (2014). Molecular Metal Catalysts on Supports: Organometallic
Chemistry Meets Surface Science. Acc. Chem.
Res..

[31] Wang L., Shao M., Xie Z.-L., Mulfort K. L. (2024). Recent Advances
in Immobilizing and Benchmarking Molecular Catalysts for Artificial
Photosynthesis. Langmuir.

[32] Copéret C., Chabanas M., Petroff
Saint-Arroman R., Basset J.-M. (2003). Homogeneous
and Heterogeneous Catalysis: Bridging the Gap through Surface Organometallic
Chemistry. Angew. Chem., Int. Ed..

[33] Kan L., Zhang L., Dong L.-Z., Wang X.-H., Li R.-H., Guo C., Li X., Yan Y., Li S.-L., Lan Y.-Q. (2024). Bridging
the Homogeneous and Heterogeneous Catalysis by Supramolecular Metal-Organic
Cages with Varied Packing Modes. Adv. Mater..

[34] Bhalothia D., Beniwal A., Kumar Saravanan P., Chen P.-C., Chen T.-Y. (2024). Bridging
the Gap Between Single Atoms, Atomic Clusters and Nanoparticles in
Electrocatalysis: Hierarchical Structured Heterogeneous Catalysts. ChemElectroChem..

[35] Sheehan S. W., Thomsen J. M., Hintermair U., Crabtree R. H., Brudvig G. W., Schmuttenmaer C. A. (2015). A Molecular
Catalyst for Water Oxidation That Binds
to Metal Oxide Surfaces. Nat. Commun..

[36] Rosser T. E., Windle C. D., Reisner E. (2016). Electrocatalytic
and Solar-Driven
CO2 Reduction to CO with a Molecular Manganese Catalyst Immobilized
on Mesoporous TiO2. Angew. Chem., Int. Ed..

[37] Schreier M., Luo J., Gao P., Moehl T., Mayer M. T., Grätzel M. (2016). Covalent Immobilization
of a Molecular Catalyst on Cu2O Photocathodes for CO2 Reduction. J. Am. Chem. Soc..

[38] Windle C. D., Kumagai H., Higashi M., Brisse R., Bold S., Jousselme B., Chavarot-Kerlidou M., Maeda K., Abe R., Ishitani O., Artero V. (2019). Earth-Abundant Molecular Z-Scheme
Photoelectrochemical Cell for Overall Water-Splitting. J. Am. Chem. Soc..

[39] Liu L., Corma A. (2018). Metal Catalysts for
Heterogeneous Catalysis: From Single Atoms to
Nanoclusters and Nanoparticles. Chem. Rev..

[40] Yang X.-F., Wang A., Qiao B., Li J., Liu J., Zhang T. (2013). Single-Atom Catalysts: A New Frontier in Heterogeneous Catalysis. Acc. Chem. Res..

[41] Yamashita H., Mori K., Shironita S., Horiuchi Y. (2008). Applications of Single-Site
Photocatalysts to the Design of Unique Surface Functional Materials. Catal. Surv. Asia.

[42] Yamashita H., Mori K. (2007). Applications of Single-Site
Photocatalysts Implanted within the Silica
Matrixes of Zeolite and Mesoporous Silica. Chem.
Lett..

[43] Li X., Bi W., Zhang L., Tao S., Chu W., Zhang Q., Luo Y., Wu C., Xie Y. (2016). Single-Atom Pt as Cocatalyst for
Enhanced Photocatalytic H2 Evolution. Adv. Mater..

[44] Xing J., Chen J. F., Li Y. H., Yuan W. T., Zhou Y., Zheng L. R., Wang H. F., Hu P., Wang Y., Zhao H. J., Wang Y., Yang H. G. (2014). Stable Isolated
Metal Atoms as Active Sites for Photocatalytic Hydrogen Evolution. Chem. – Eur. J..

[45] Jeantelot G., Qureshi M., Harb M., Ould-Chikh S., Anjum D. H., Abou-Hamad E., Aguilar-Tapia A., Hazemann J.-L., Takanabe K., Basset J.-M. (2019). TiO2-Supported Pt
Single Atoms by Surface Organometallic Chemistry for Photocatalytic
Hydrogen Evolution. Phys. Chem. Chem. Phys..

[46] Gao G., Jiao Y., Waclawik E. R., Du A. (2016). Single Atom (Pd/Pt)
Supported on Graphitic Carbon Nitride as an Efficient Photocatalyst
for Visible-Light Reduction of Carbon Dioxide. J. Am. Chem. Soc..

[47] Liu W., Cao L., Cheng W., Cao Y., Liu X., Zhang W., Mou X., Jin L., Zheng X., Che W., Liu Q., Yao T., Wei S. (2017). Single-Site Active Cobalt-Based Photocatalyst with
a Long Carrier Lifetime for Spontaneous Overall Water Splitting. Angew. Chem., Int. Ed..

[48] Neubert S., Mitoraj D., Shevlin S. A., Pulisova P., Heimann M., Du Y., Goh G. K. L., Pacia M., Kruczała K., Turner S., Macyk W., Guo Z. X., Hocking R. K., Beranek R. (2016). Highly Efficient Rutile
TiO2 Photocatalysts with Single
Cu­(II) and Fe­(III) Surface Catalytic Sites. J. Mater. Chem. A.

[49] Gao C., Low J., Long R., Kong T., Zhu J., Xiong Y. (2020). Heterogeneous
Single-Atom Photocatalysts: Fundamentals and Applications. Chem. Rev..

[50] Yadav V., Jana A., Acharya S., Malola S., Nagar H., Sharma A., Kini A. R., Antharjanam S., Machacek J., Adarsh K. N. V. D., Base T., Häkkinen H., Pradeep T. (2025). Site-Specific Substitution
in Atomically Precise Carboranethiol-Protected
Nanoclusters and Concomitant Changes in Electronic Properties. Nat. Commun..

[51] Li X., Mitchell S., Fang Y., Li J., Perez-Ramirez J., Lu J. (2023). Advances in Heterogeneous Single-Cluster Catalysis. Nat. Rev. Chem..

[52] Aniceto-Ocaña P., Marqueses-Rodriguez J., Perez-Omil J. A., Calvino J. J., Castillo C. E., Lopez-Haro M. (2024). Direct Quantitative
Assessment of Single-Atom Metal
Sites Supported on Powder Catalysts. Commun.
Mater..

[53] Mielke J., Hanke F., Peters M. V., Hecht S., Persson M., Grill L. (2015). Adatoms underneath Single Porphyrin Molecules on Au(111). J. Am. Chem. Soc..

[54] Li S., Lin Y.-C., Zhao W., Wu J., Wang Z., Hu Z., Shen Y., Tang D.-M., Wang J., Zhang Q., Zhu H., Chu L., Zhao W., Liu C., Sun Z., Taniguchi T., Osada M., Chen W., Xu Q.-H., Wee A. T. S., Suenaga K., Ding F., Eda G. (2018). Vapour–Liquid–Solid
Growth of Monolayer MoS2 Nanoribbons. Nat. Mater..

[55] Nguyen H. A., Hammel B. F., Sharp D., Kline J., Schwartz G., Harvey S., Nishiwaki E., Sandeno S. F., Ginger D. S., Majumdar A., Yazdi S., Dukovic G., Cossairt B. M. (2024). Colossal
Core/Shell CdSe/CdS Quantum Dot Emitters. ACS
Nano.

[56] An H., Yan X., Li H., Yang B., Wei J., Yang G. (2019). Increased
Active Sites by in Situ Growth of CoP Quantum Dots on CdS/rGO To Achieve
Efficient Photocatalytic H2 Production. ACS
Appl. Energy Mater..

[57] Liu J., Liu Y., Liu N., Han Y., Zhang X., Huang H., Lifshitz Y., Lee S.-T., Zhong J., Kang Z. (2015). Metal-Free
Efficient Photocatalyst for Stable Visible Water Splitting via a Two-Electron
Pathway. Science.

[58] Tan W., Li Y., Jiang W., Gao C., Zhuang C. (2020). CdS Nanospheres Decorated
with NiS Quantum Dots as Nobel-Metal-Free Photocatalysts for Efficient
Hydrogen Evolution. ACS Appl. Energy Mater..

[59] Hazra M., Hofer M., Fickl B., Ertl A., Myakala S. N., Eder D., Porcu S., Cherevan A., Bayer B. C., Ricci P. C. (2025). 2D-2D PhCN/WS2 Exfoliated Nanosheets for Visible-Light
Hydrogen Production: A Platinum-Free Cocatalyst Approach. Carbon.

[60] Lian K., Yue Y., Basset J.-M., Liu X., Chen L., Ozsoy-Keskinbora C., Bao X., Zhu H. (2022). Surface Organometallic
Chemistry as a Versatile Strategy
for Synthesizing Supported Bimetallic Cluster Catalysts. J. Phys. Chem. C.

[61] Müller A., Beckmann E., Bögge H., Schmidtmann M., Dress A. (2002). Inorganic Chemistry Goes Protein
Size: A Mo368 Nano-Hedgehog Initiating
Nanochemistry by Symmetry Breaking. Angew. Chem.,
Int. Ed..

[62] Guo J., Xue X., Yu H., Duan Y., Li F., Lian Y., Liu Y., Zhao M. (2022). Metal–Organic Frameworks Based on Infinite Secondary
Building Units: Recent Progress and Future Outlooks. J. Mater. Chem. A.

[63] Ayala P., Naghdi S., Nandan S. P., Myakala S. N., Rath J., Saito H., Guggenberger P., Lakhanlal L., Kleitz F., Toroker M. C., Cherevan A., Eder D. (2023). The Emergence
of 2D Building Units in Metal-Organic Frameworks for Photocatalytic
Hydrogen Evolution: A Case Study with COK-47. Adv. Energy Mater..

[64] Müller A., Sarkar S., Bhattacharyya R. G., Pohl S., Dartmann M. (1978). Directed Synthesis
of [Mo3S13]­2–, an Isolated Cluster Containing Sulfur Atoms
in Three Different States of Bonding. Angew.
Chem., Int. Ed. Engl..

[65] Müller A., Nolte W.-O., Krebs B. (1978). [(S2)­2Mo­(S2)­2Mo­(S2)­2]­2–,
a
Novel Complex Containing Only S Ligands and a MoMo Bond. Angew. Chem., Int. Ed. Engl..

[66] Batool S., Langer M., Myakala S. N., Heiland M., Eder D., Streb C., Cherevan A. (2024). Thiomolybdate
Clusters: From Homogeneous
Catalysis to Heterogenization and Active Sites. Adv. Mater..

[67] Jaramillo T. F., Bonde J., Zhang J., Ooi B.-L., Andersson K., Ulstrup J., Chorkendorff I. (2008). Hydrogen Evolution on Supported Incomplete
Cubane-Type [Mo3S4]­4+ Electrocatalysts. J. Phys.
Chem. C.

[68] Hellstern T. R., Kibsgaard J., Tsai C., Palm D. W., King L. A., Abild-Pedersen F., Jaramillo T. F. (2017). Investigating Catalyst–Support
Interactions To Improve the Hydrogen Evolution Reaction Activity of
Thiomolybdate [Mo3S13]­2– Nanoclusters. ACS Catal..

[69] Seo B., Jung G. Y., Lee S. J., Baek D. S., Sa Y. J., Ban H. W., Son J. S., Park K., Kwak S. K., Joo S. H. (2020). Monomeric MoS42–-Derived Polymeric Chains with
Active Molecular Units for Efficient Hydrogen Evolution Reaction. ACS Catal..

[70] Huang Z., Luo W., Ma L., Yu M., Ren X., He M., Polen S., Click K., Garrett B., Lu J., Amine K., Hadad C., Chen W., Asthagiri A., Wu Y. (2015). Dimeric [Mo2S12]­2– Cluster: A Molecular Analogue of MoS2 Edges
for Superior Hydrogen-Evolution Electrocatalysis. Angew. Chem., Int. Ed..

[71] McAllister J., Bandeira N. A. G., McGlynn J. C., Ganin A. Y., Song Y.-F., Bo C., Miras H. N. (2019). Tuning
and Mechanistic Insights of Metal Chalcogenide
Molecular Catalysts for the Hydrogen-Evolution Reaction. Nat. Commun..

[72] Dave M., Rajagopal A., Damm-Ruttensperger M., Schwarz B., Nägele F., Daccache L., Fantauzzi D., Jacob T., Streb C. (2018). Understanding
Homogeneous Hydrogen Evolution Reactivity and Deactivation Pathways
of Molecular Molybdenum Sulfide Catalysts. Sustain.
Energy Fuels.

[73] Lei Y., Yang M., Hou J., Wang F., Cui E., Kong C., Min S. (2018). Thiomolybdate [Mo3S13]­2– Nanocluster:
A Molecular Mimic of MoS2 Active Sites for Highly Efficient Photocatalytic
Hydrogen Evolution. Chem. Commun..

[74] Rajagopal A., Venter F., Jacob T., Petermann L., Rau S., Tschierlei S., Streb C. (2019). Homogeneous Visible Light-Driven
Hydrogen Evolution by the Molecular Molybdenum Sulfide Model [Mo2S12]­2–. Sustain. Energy Fuels.

[75] Baloglou A., Plattner M., Ončák M., Grutza M.-L., Kurz P., Beyer M. K. (2021). [Mo3S13]­2–
as a Model System for Hydrogen Evolution
Catalysis by MoSx: Probing Protonation Sites in the Gas Phase by Infrared
Multiple Photon Dissociation Spectroscopy. Angew.
Chem., Int. Ed..

[76] Baloglou A., Ončák M., Grutza M.-L., van der Linde C., Kurz P., Beyer M. K. (2019). Structural
Properties of Gas Phase
Molybdenum Sulfide Clusters [Mo3S13]­2–, [HMo3S13]–,
and [H3Mo3S13]+ as Model Systems of a Promising Hydrogen Evolution
Catalyst. J. Phys. Chem. C.

[77] Tran P. D., Tran T. V., Orio M., Torelli S., Truong Q. D., Nayuki K., Sasaki Y., Chiam S. Y., Yi R., Honma I., Barber J., Artero V. (2016). Coordination Polymer
Structure and Revisited Hydrogen Evolution Catalytic Mechanism for
Amorphous Molybdenum Sulfide. Nat. Mater..

[78] Bau J. A., Emwas A.-H., Nikolaienko P., Aljarb A. A., Tung V., Rueping M. (2022). Mo3+ Hydride as the
Common Origin of H2 Evolution and
Selective NADH Regeneration in Molybdenum Sulfide Electrocatalysts. Nat. Catal..

[79] Bau J. A., Ahmad R., Cavallo L., Rueping M. (2022). A Unified
Theory for
H2 Evolution on Mo-Based Electrocatalysts. ACS
Energy Lett..

[80] Recatalá D., Llusar R., Gushchin A. L., Kozlova E. A., Laricheva Y. A., Abramov P. A., Sokolov M. N., Gómez R., Lana-Villarreal T. (2015). Photogeneration of Hydrogen from Water by Hybrid Molybdenum
Sulfide Clusters Immobilized on Titania. ChemSusChem.

[81] Batool S., Nandan S. P., Myakala S. N., Rajagopal A., Schubert J. S., Ayala P., Naghdi S., Saito H., Bernardi J., Streb C., Cherevan A., Eder D. (2022). Surface Anchoring
and Active Sites of [Mo3S13]­2– Clusters as Cocatalysts for
Photocatalytic Hydrogen Evolution. ACS Catal..

[82] Zhang R., Gong K., Du F., Cao S. (2022). Highly Efficient Thiomolybdate
[Mo2S12]­2- Nanocluster Cocatalyst Decorated on TiO2 to Boost Photocatalytic
Hydrogen Evolution. Int. J. Hydrog. Energy.

[83] Guo F., Hou Y., Asiri A. M., Wang X. (2017). Assembly of Protonated Mesoporous
Carbon Nitrides with Cocatalytic [Mo3S13]­2– Clusters for Photocatalytic
Hydrogen Production. Chem. Commun..

[84] Batool S., Schubert J. S., Ayala P., Saito H., Sampaio M. J., Da Silva E. S., Silva C. G., Faria J. L., Eder D., Cherevan A. (2024). A Thiomolybdate Cluster
for Visible-Light-Driven Hydrogen
Evolution: Comparison of Homogeneous and Heterogeneous Approaches. Sustain. Energy Fuels.

[85] Rajagopal A., Akbarzadeh E., Li C., Mitoraj D., Krivtsov I., Adler C., Diemant T., Biskupek J., Kaiser U., Im C., Heiland M., Jacob T., Streb C., Dietzek B., Beranek R. (2020). Polymeric Carbon Nitride Coupled with a Molecular Thiomolybdate
Catalyst: Exciton and Charge Dynamics in Light-Driven Hydrogen Evolution. Sustain. Energy Fuels.

[86] Benck J. D., Lee S. C., Fong K. D., Kibsgaard J., Sinclair R., Jaramillo T. F. (2014). Designing
Active and Stable Silicon
Photocathodes for Solar Hydrogen Production Using Molybdenum Sulfide
Nanomaterials. Adv. Energy Mater..

[87] Bozheyev F., Xi F., Plate P., Dittrich T., Fiechter S., Ellmer K. (2019). Efficient
Charge Transfer at a Homogeneously Distributed (NH4)­2Mo3S13/WSe2 Heterojunction
for Solar Hydrogen Evolution. J. Mater. Chem.
A.

[88] Xi F., Bozheyev F., Han H., Rusu M., Rappich J., Abdi F. F., Bogdanoff P., Kaltsoyannis N., Fiechter S. (2022). Enhancing Hydrogen Evolution Reaction via Synergistic
Interaction between the [Mo3S13]­2– Cluster Cocatalyst and WSe2
Photocathode. ACS Appl. Mater. Interfaces.

[89] Bozheyev F., Fengler S., Kollmann J., Klassen T., Schieda M. (2022). Transient
Surface Photovoltage Spectroscopy of (NH4)­2Mo3S13/WSe2 Thin-Film Photocathodes
for Photoelectrochemical Hydrogen Evolution. ACS Appl. Mater. Interfaces.

[90] Barros
Barbosa J., Taberna P. L., Bourdon V., Gerber I. C., Poteau R., Balocchi A., Marie X., Esvan J., Puech P., Barnabé A., Da Gama Fernandes Vieira L., Moraru I.-T., Chane-Ching J. Y. (2020). Mo Thio and Oxo-Thio Molecular Complexes
Film as Self-Healing Catalyst for Photocatalytic Hydrogen Evolution
on 2D Materials. Appl. Catal. B Environ..

[91] Adams P., Bühler J., Walz I., Moehl T., Roithmeyer H., Blacque O., Comini N., Diulus J. T., Alberto R., Siol S., Dimitrievska M., Novotny Z., Tilley S. D. (2024). Direct
Anchoring of Molybdenum Sulfide Molecular Catalysts on Antimony Selenide
Photocathodes for Solar Hydrogen Production. ACS Energy Lett..

[92] Gray M., Song Y. F., Miras H. N. (2025). Heterogenization
of Molecular Chalcoxides
for Electro-& Photochemical H 2 Production. Chem. Commun..

[93] Hill C. L., Bouchard D. A. (1985). Catalytic Photochemical Dehydrogenation of Organic
Substrates by Polyoxometalates. J. Am. Chem.
Soc..

[94] Pope M. T., Müller A. (1991). Polyoxometalate Chemistry: An Old Field with New Dimensions
in Several Disciplines. Angew. Chem., Int. Ed.
Engl..

[95] Katsoulis D. E. (1998). A Survey
of Applications of Polyoxometalates. Chem. Rev..

[96] Kozhevnikov I. V. (1998). Catalysis
by Heteropoly Acids and Multicomponent Polyoxometalates in Liquid-Phase
Reactions. Chem. Rev..

[97] Streb C. (2012). New Trends
in Polyoxometalate Photoredox Chemistry: From Photosensitisation to
Water Oxidation Catalysis. Dalton Trans..

[98] Wang S.-S., Yang G.-Y. (2015). Recent Advances
in Polyoxometalate-Catalyzed Reactions. Chem.
Rev..

[99] Meng R.-Q., Suo L., Hou G.-F., Liang J., Bi L.-H., Li H.-L., Wu L.-X. (2013). Organo-Ru Supported Sandwich-Type Tungstoarsenates: Synthesis. Structure and Catalytic Properties. CrystEngComm.

[100] Sartorel A., Carraro M., Scorrano G., Zorzi R. D., Geremia S., McDaniel N. D., Bernhard S., Bonchio M. (2008). Polyoxometalate
Embedding of a Tetraruthenium­(IV)-Oxo-Core by Template-Directed Metalation
of [γ-SiW10O36]­8–: A Totally Inorganic Oxygen-Evolving
Catalyst. J. Am. Chem. Soc..

[101] Geletii Y. V., Huang Z., Hou Y., Musaev D. G., Lian T., Hill C. L. (2009). Homogeneous Light-Driven
Water Oxidation
Catalyzed by a Tetraruthenium Complex with All Inorganic Ligands. J. Am. Chem. Soc..

[102] Geletii Y. V., Besson C., Hou Y., Yin Q., Musaev D. G., Quiñonero D., Cao R., Hardcastle K. I., Proust A., Kögerler P., Hill C. L. (2009). Structural, Physicochemical,
and Reactivity Properties of an All-Inorganic, Highly Active Tetraruthenium
Homogeneous Catalyst for Water Oxidation. J.
Am. Chem. Soc..

[103] Yin Q., Tan J. M., Besson C., Geletii Y. V., Musaev D. G., Kuznetsov A. E., Luo Z., Hardcastle K. I., Hill C. L. (2010). A Fast Soluble Carbon-Free
Molecular Water Oxidation
Catalyst Based on Abundant Metals. Science.

[104] Al-Sayed E., Nandan S. P., Tanuhadi E., Giester G., Arrigoni M., Madsen G. K. H., Cherevan A., Eder D., Rompel A. (2021). Phosphate-Templated
Encapsulation of a CoII4O4 Cubane
in Germanotungstates as Carbon-Free Homogeneous Water Oxidation Photocatalysts. ChemSusChem.

[105] Tanuhadi E., Cano J., Batool S., Cherevan A., Eder D., Rompel A. (2022). Ni12 Tetracubane Cores
with Slow
Relaxation of Magnetization and Efficient Charge Utilization for Photocatalytic
Hydrogen Evolution. J. Mater. Chem. C.

[106] Cherevan A. S., Nandan S. P., Roger I., Liu R., Streb C., Eder D. (2020). Polyoxometalates on Functional Substrates:
Concepts, Synergies, and Future Perspectives. Adv. Sci..

[107] Kato C. N., Hara K., Hatano A., Goto K., Kuribayashi T., Hayashi K., Shinohara A., Kataoka Y., Mori W., Nomiya K. (2008). A Dawson-Type Dirhenium­(V)-Oxido-Bridged
Polyoxotungstate: X-Ray Crystal Structure and Hydrogen Evolution from
Water Vapor under Visible Light Irradiation. Eur. J. Inorg. Chem..

[108] Song F., Ding Y., Ma B., Wang C., Wang Q., Du X., Fu S., Song J. (2013). K7­[CoIIICoII­(H2O)­W11O39]:
A Molecular Mixed-Valence Keggin Polyoxometalate Catalyst of High
Stability and Efficiency for Visible Light-Driven Water Oxidation. Energy Environ. Sci..

[109] Nandan S. P., Gumerova N. I., Schubert J. S., Saito H., Rompel A., Cherevan A., Eder D. (2022). Immobilization
of a
[CoIIICoII­(H2O)­W11O39]­7– Polyoxoanion for the Photocatalytic
Oxygen Evolution Reaction. ACS Mater. Au.

[110] Kato C. N., Morii Y., Hattori S., Nakayama R., Makino Y., Uno H. (2012). Diplatinum­(II)-Coordinated Polyoxotungstate:
Synthesis, Molecular Structure, and Photocatalytic Performance for
Hydrogen Evolution from Water under Visible-Light Irradiation. Dalton Trans..

[111] Cao Y.-D., Yin D., Wang M.-L., Pang T., Lv Y., Liu B., Gao G.-G., Ma L., Liu H. (2020). Pt-Substituted
Polyoxometalate Modification on the Surface of Low-Cost TiO2 with
Highly Efficient H2 Evolution Performance. Dalton
Trans..

[112] Zhou X., Yu H., Zhao D., Wang X., Zheng S. (2019). Combination of Polyoxotantalate
and Metal Sulfide: A New-Type Noble-Metal-Free
Binary Photocatalyst Na8Ta6O19/Cd0.7Zn0.3S for Highly Efficient Visible-Light-Driven
H2 Evolution. Appl. Catal. B Environ..

[113] Xing X., Liu R., Yu X., Zhang G., Cao H., Yao J., Ren B., Jiang Z., Zhao H. (2013). Self-Assembly
of CdS Quantum Dots with Polyoxometalate Encapsulated Gold Nanoparticles:
Enhanced Photocatalytic Activities. J. Mater.
Chem. A.

[114] Dong Y., Hu Q., Li B., Li X., Chen M., Zhang M., Feng Y., Ding Y. (2022). Aminated Silicon
Dioxide Enriching Iron-Containing Polyoxometalate Catalyst Confined
in CdS for Efficient H2 Evolution. Appl. Catal.
B Environ..

[115] Du X., Zhao J., Mi J., Ding Y., Zhou P., Ma B., Zhao J., Song J. (2015). Efficient Photocatalytic H2 Evolution
Catalyzed by an Unprecedented Robust Molecular Semiconductor {Fe11}
Nanocluster without Cocatalysts at Neutral Conditions. Nano Energy.

[116] Zhang Z.-M., Zhang T., Wang C., Lin Z., Long L.-S., Lin W. (2015). Photosensitizing Metal–Organic
Framework Enabling Visible-Light-Driven Proton Reduction by a Wells–Dawson-Type
Polyoxometalate. J. Am. Chem. Soc..

[117] Jiao L., Dong Y., Xin X., Qin L., Lv H. (2021). Facile Integration
of Ni-Substituted Polyoxometalate Catalysts into
Mesoporous Light-Responsive Metal-Organic Framework for Effective
Photogeneration of Hydrogen. Appl. Catal. B
Environ..

[118] Glasby L. T., Gubsch K., Bence R., Oktavian R., Isoko K., Moosavi S. M., Cordiner J. L., Cole J. C., Moghadam P. Z. (2023). DigiMOF:
A Database of Metal–Organic Framework
Synthesis Information Generated via Text Mining. Chem. Mater..

[119] Li H., Eddaoudi M., O’Keeffe M., Yaghi O. M. (1999). Design and Synthesis
of an Exceptionally Stable and Highly Porous Metal-Organic Framework. Nature.

[120] Gordon R. M., Silver H. B. (1983). Preparation and
Properties of Tetrazinc
Μ4-Oxohexa-μ-Carboxylates (Basic Zinc Carboxylates). Can. J. Chem..

[121] Clegg W., Harbron D. R., Homan C. D., Hunt P. A., Little I. R., Straughan B. P. (1991). Crystal
Structures of Three Basic
Zinc Carboxylates Together with Infrared and FAB Mass Spectrometry
Studies in Solution. Inorg. Chim. Acta.

[122] Koyama H., Saito Y. (1954). The Crystal Structure of Zinc Oxyacetate,
Zn4O­(CH3COO)­6. Bull. Chem. Soc. Jpn..

[123] Bury W., Justyniak I., Prochowicz D., Wróbel Z., Lewiński J. (2012). Oxozinc Carboxylates: A Predesigned
Platform for Modelling Prototypical Zn-MOFs’ Reactivity toward
Water and Donor Solvents. Chem. Commun..

[124] Terlecki M., Justyniak I., Leszczyński M.
K., Lewiński J. (2021). Effect of
the Proximal Secondary Sphere on the Self-Assembly
of Tetrahedral Zinc-Oxo Clusters. Commun. Chem..

[125] Férey G., Mellot-Draznieks C., Serre C., Millange F., Dutour J., Surblé S., Margiolaki I. (2005). A Chromium
Terephthalate-Based Solid with Unusually Large Pore Volumes and Surface
Area. Science.

[126] Cannon, R. D. ; White, R. P. Chemical and Physical Properties of Triangular Bridged Metal Complexes. In Prog. Inorg. Chem.; John Wiley & Sons, Ltd, 1988; pp 195–298. 10.1002/9780470166376.ch3.

[127] Figuerola A., Tangoulis V., Ribas J., Hartl H., Brüdgam I., Maestro M., Diaz C. (2007). Synthesis, Crystal
Structure, and Magnetic Studies of Oxo-Centered Trinuclear Chromium­(III)
Complexes: [Cr3­(Μ3-O)­(Μ2-PhCOO)­6­(H2O)­3]­NO3· 4H2O·2CH3OH,
a Case of Spin-Frustrated System, and [Cr3­(Μ3-O)- (Μ2-PhCOO)­2­(Μ2-OCH2CH3)­2­(Bpy)­2­(NCS)­3],
a New Type of [Cr3O] Core. Inorg. Chem..

[128] Peng L., Asgari M., Mieville P., Schouwink P., Bulut S., Sun D. T., Zhou Z., Pattison P., van Beek W., Queen W. L. (2017). Using Predefined
M3­(Μ3-O) Clusters
as Building Blocks for an Isostructural Series of Metal–Organic
Frameworks. ACS Appl. Mater. Interfaces.

[129] Shearer G. C., Chavan S., Bordiga S., Svelle S., Olsbye U., Lillerud K. P. (2016). Defect Engineering: Tuning the Porosity
and Composition of the Metal–Organic Framework UiO-66 via Modulated
Synthesis. Chem. Mater..

[130] Schubert U. (2022). Clusters with a Zr6O8 Core. Coord.
Chem. Rev..

[131] Kickelbick G., Schubert U. (1997). Oxozirconium Methacrylate Clusters:
Zr6­(OH)­4O4­(OMc)­12 and Zr4O2­(OMc)­12 (OMc = Methacrylate). Chem. Ber..

[132] Gao Y., Kogler F. R., Peterlik H., Schubert U. (2006). Ring-Opening
Metathesis
Polymerizations with Norbornene Carboxylate-Substituted Metal Oxo
Clusters. J. Mater. Chem..

[133] Pascual-Colino J., Artetxe B., Beobide G., Castillo O., Fidalgo-Mayo M. L., Isla-López A., Luque A., Mena-Gutiérrez S., Pérez-Yáñez S. (2022). The Chemistry
of Zirconium/Carboxylate
Clustering Process: Acidic Conditions to Promote Carboxylate-Unsaturated
Octahedral Hexamers and Pentanuclear Species. Inorg. Chem..

[134] Yang Y., Broto-Ribas A., Ortín-Rubio B., Imaz I., Gándara F., Carné-Sánchez A., Guillerm V., Jurado S., Busqué F., Juanhuix J., Maspoch D. (2022). Clip-off Chemistry: Synthesis by
Programmed Disassembly of Reticular Materials. Angew. Chem., Int. Ed..

[135] Ruiz-Relaño S., Nam D., Albalad J., Cortés-Martínez A., Juanhuix J., Imaz I., Maspoch D. (2024). Synthesis of Metal–Organic
Cages via Orthogonal Bond Cleavage in 3D Metal–Organic Frameworks. J. Am. Chem. Soc..

[136] Sánchez-Naya R., Cavalieri J. P., Albalad J., Cortés-Martínez A., Wang K., Fuertes-Espinosa C., Parella T., Fiori S., Ribas E., Mugarza A., Ribas X., Faraudo J., Yaghi O. M., Imaz I., Maspoch D. (2025). Excision of Organic
Macrocycles from Covalent Organic Frameworks. Science.

[137] Tranchemontagne D. J., Ni Z., O’Keeffe M., Yaghi O. M. (2008). Reticular Chemistry of Metal–Organic Polyhedra. Angew. Chem., Int. Ed..

[138] Chen S., Li K., Zhao F., Zhang L., Pan M., Fan Y.-Z., Guo J., Shi J., Su C.-Y. (2016). A Metal-Organic
Cage Incorporating Multiple Light Harvesting and Catalytic Centres
for Photochemical Hydrogen Production. Nat.
Commun..

[139] Vardhan H., Verpoort F. (2015). Metal-Organic Polyhedra: Catalysis
and Reactive Intermediates. Adv. Synth. Catal..

[140] Sun M., Wang Q., Qin C., Sun C., Wang X., Su Z. (2019). An Amine-Functionalized Zirconium Metal–Organic Polyhedron
Photocatalyst with High Visible-Light Activity for Hydrogen Production. Chem. – Eur. J..

[141] Ghosh A. C., Legrand A., Rajapaksha R., Craig G. A., Sassoye C., Balázs G., Farrusseng D., Furukawa S., Canivet J., Wisser F. M. (2022). Rhodium-Based
Metal–Organic Polyhedra Assemblies for Selective CO2 Photoreduction. J. Am. Chem. Soc..

[142] Kang Y.-H., Liu X.-D., Yan N., Jiang Y., Liu X.-Q., Sun L.-B., Li J.-R. (2016). Fabrication
of Isolated
Metal–Organic Polyhedra in Confined Cavities: Adsorbents/Catalysts
with Unusual Dispersity and Activity. J. Am.
Chem. Soc..

[143] Ham R., Nielsen C. J., Pullen S., Reek J. N. (2023). Supramolecular Coordination
Cages for Artificial Photosynthesis and Synthetic Photocatalysis. Chem. Rev..

[144] Qin S., Lei Y., Huang J., Xiao L., Liu J. (2021). A Robust Photocatalytic
Hybrid Material Composed of Metal-Organic Cages and TiO2 for Efficient
Visible-Light-Driven Hydrogen Evolution. Chem.
– Asian J..

[145] Qin S., Lei Y., Guo J., Huang J.-F., Hou C.-P., Liu J.-M. (2021). Constructing Heterogeneous Direct Z-Scheme Photocatalysts
Based on Metal–Organic Cages and Graphitic-C3N4 for High-Efficiency
Photocatalytic Water Splitting. ACS Appl. Mater.
Interfaces.

[146] Liang Z.-Z., Li X.-A., Chen Q.-Z., Wang X.-L., Su P.-Y., Huang J.-F., Zhou Y., Xiao L.-M., Liu J.-M. (2024). A Direct Z-Scheme Single-Atom MOC/COF Piezo-Photocatalytic
System for Overall Water Splitting. ACS Catal..

[147] Hong K., Chun H. (2013). Nonporous Titanium–Oxo Molecular
Clusters That Reversibly and Selectively Adsorb Carbon Dioxide. Inorg. Chem..

[148] Zhou J., Li J., Kan L., Zhang L., Huang Q., Yan Y., Chen Y., Liu J., Li S.-L., Lan Y.-Q. (2022). Linking Oxidative and Reductive Clusters
to Prepare Crystalline Porous Catalysts for Photocatalytic CO2 Reduction
with H2O. Nat. Commun..

[149] Nohra B., El Moll H., Rodriguez
Albelo L. M., Mialane P., Marrot J., Mellot-Draznieks C., O’Keeffe M., Ngo Biboum R., Lemaire J., Keita B., Nadjo L., Dolbecq A. (2011). Polyoxometalate-Based Metal Organic
Frameworks (POMOFs): Structural Trends, Energetics, and High Electrocatalytic
Efficiency for Hydrogen Evolution Reaction. J. Am. Chem. Soc..

[150] Amthor S., Knoll S., Heiland M., Zedler L., Li C., Nauroozi D., Tobaschus W., Mengele A. K., Anjass M., Schubert U. S., Dietzek-Ivanšić B., Rau S., Streb C. (2022). A Photosensitizer–Polyoxometalate Dyad That
Enables the Decoupling of Light and Dark Reactions for Delayed on-Demand
Solar Hydrogen Production. Nat. Chem..

[151] Maloul S., van den Borg M., Müller C., Zedler L., Mengele A. K., Gaissmaier D., Jacob T., Rau S., Dietzek-Ivanšič B., Streb C. (2021). Multifunctional Polyoxometalate Platforms for Supramolecular Light-Driven
Hydrogen Evolution. Chem. – Eur. J..

[152] Gumerova N. I., Rompel A. (2018). Synthesis, Structures
and Applications
of Electron-Rich Polyoxometalates. Nat. Rev.
Chem..

[153] Li X.-X., Zhao D., Zheng S.-T. (2019). Recent
Advances
in POM-Organic Frameworks and POM-Organic Polyhedra. Coord. Chem. Rev..

[154] Tomita O., Naito H., Nakada A., Higashi M., Abe R. (2022). Mono-Transition-Metal-Substituted Polyoxometalates as Shuttle Redox
Mediator for Z-Scheme Water Splitting under Visible Light. Sustain. Energy Fuels.

[155] Chen W., Li H., Jin Y., Lei W., Bai Q., Ma P., Wang J., Niu J. (2023). Construction
of Hexameric
Ru-Substitution POMs to Improve Photocatalytic H2 Evolution. Inorg. Chem..

[156] Zhang S., Sun Y., Chen W., Li H., Li K., Ma P., Wang J., Niu J. (2025). Facile Synthesis
of
a Ru-Based Polyoxometalate for Efficient Reduction of Nitrobenzene. Dalton Trans..

[157] Booth G., Chatt J., Chini P. (1965). Platinum Carbonyls
Substituted by Tertiary Phosphines. Chem. Commun.
London.

[158] Du Y., Sheng H., Astruc D., Zhu M. (2020). Atomically Precise
Noble Metal Nanoclusters as Efficient Catalysts: A Bridge between
Structure and Properties. Chem. Rev..

[159] Chu K., Luo Y., Wu D., Su Z., Shi J., Zhang J. Z., Su C.-Y. (2021). Charge State of Au25­(SG)­18 Nanoclusters
Induced by Interaction with a Metal Organic Framework Support and
Its Effect on Catalytic Performance. J. Phys.
Chem. Lett..

[160] Kawawaki T., Kataoka Y., Ozaki S., Kawachi M., Hirata M., Negishi Y. (2021). Creation of Active Water-Splitting
Photocatalysts by Controlling Cocatalysts Using Atomically Precise
Metal Nanoclusters. Chem. Commun..

[161] Liu Y., Wang Y., Pinna N. (2024). Atomically
Precise Metal Nanoclusters
for Photocatalytic Water Splitting. ACS Mater.
Lett..

[162] Liang H., Liu B.-J., Tang B., Zhu S.-C., Li S., Ge X.-Z., Li J.-L., Zhu J.-R., Xiao F.-X. (2022). Atomically
Precise Metal Nanocluster-Mediated Photocatalysis. ACS Catal..

[163] Wang Y., Liu X.-H., Wang Q., Quick M., Kovalenko S. A., Chen Q.-Y., Koch N., Pinna N. (2020). Insights into
Charge Transfer at an Atomically Precise Nanocluster/Semiconductor
Interface. Angew. Chem., Int. Ed..

[164] Zou X., Zhang Y. (2015). Noble Metal-Free Hydrogen Evolution Catalysts for Water
Splitting. Chem. Soc. Rev..

[165] Zeng H., Ji Y., Wen J., Li X., Zheng T., Jiang Q., Xia C. (2025). Pt Nanocluster-Catalyzed
Hydrogen Evolution Reaction: Recent Advances and Future Outlook. Chin. Chem. Lett..

[166] Schweinberger F. F., Berr M. J., Döblinger M., Wolff C., Sanwald K. E., Crampton A. S., Ridge C. J., Jäckel F., Feldmann J., Tschurl M., Heiz U. (2013). Cluster Size
Effects in the Photocatalytic Hydrogen Evolution Reaction. J. Am. Chem. Soc..

[167] Liu Y., Long D., Springer A., Wang R., Koch N., Schwalbe M., Pinna N., Wang Y. (2023). Correlating Heteroatoms
Doping, Electronic Structures, and Photocatalytic Activities of Single-Atom-Doped
Ag25­(SR)­18 Nanoclusters. Sol. RRL.

[168] Lu X., Tong A., Luo D., Jiang F., Wei J., Huang Y., Jiang Z., Lu Z., Ni Y. (2022). Confining
Single Pt Atoms from Pt Clusters on Multi-Armed CdS for Enhanced Photocatalytic
Hydrogen Evolution. J. Mater. Chem. A.

[169] Li S., Du X., Liu Z., Li Y., Shao Y., Jin R. (2023). Size Effects of Atomically
Precise Gold Nanoclusters in Catalysis. Precis.
Chem..

[170] Ling S., Cui X., Zhang X., Liu B., He C., Wang J., Qin W., Zhang Y., Gao Y., Bai G. (2018). Glutathione-Protected Gold Nanocluster Decorated Cadmium Sulfide
with Enhanced Photostability and Photocatalytic Activity. J. Colloid Interface Sci..

[171] Zhao S., Jin R., Song Y., Zhang H., House S. D., Yang J. C., Jin R. (2017). Atomically
Precise
Gold Nanoclusters Accelerate Hydrogen Evolution over MoS2 Nanosheets:
The Dual Interfacial Effect. Small.

[172] Shen P., Zhao S., Su D., Li Y., Orlov A. (2012). Outstanding Activity of Sub-Nm Au Clusters for Photocatalytic
Hydrogen
Production. Appl. Catal. B Environ..

[173] Negishi Y., Mizuno M., Hirayama M., Omatoi M., Takayama T., Iwase A., Kudo A. (2013). Enhanced Photocatalytic
Water Splitting by BaLa4Ti4O15 Loaded with ∼ 1 Nm Gold Nanoclusters
Using Glutathione-Protected Au25 Clusters. Nanoscale.

[174] Du X. L., Wang X. L., Li Y. H., Wang Y. L., Zhao J. J., Fang L. J., Zheng L. R., Tong H., Yang H. G. (2017). Isolation of Single
Pt Atoms in a Silver Cluster: Forming
Highly Efficient Silver-Based Cocatalysts for Photocatalytic Hydrogen
Evolution. Chem. Commun..

[175] Bootharaju M. S., Lee C. W., Deng G., Kim H., Lee K., Lee S., Chang H., Lee S., Sung Y.-E., Yoo J. S., Zheng N., Hyeon T. (2023). Atom-Precise Heteroatom
Core-Tailoring of Nanoclusters for Enhanced Solar Hydrogen Generation. Adv. Mater..

[176] Wang H., Zhang X., Zhang W., Zhou M., Jiang H.-L. (2024). Heteroatom-Doped Ag25 Nanoclusters Encapsulated in
Metal–Organic Frameworks for Photocatalytic Hydrogen Production. Angew. Chem., Int. Ed..

[177] Tan D., Ding T., Shen K., Xu C., Jin S., Hu D., Sun S., Zhu M. (2025). Icosahedron
Kernel Defect in Pt1Agx
Series of Bimetallic Nanoclusters Enhances Photocatalytic Hydrogen
Evolution. Chem. Sci..

[178] Kagalwala H. N., Gottlieb E., Li G., Li T., Jin R., Bernhard S. (2013). Photocatalytic Hydrogen Generation
System Using a Nickel-Thiolate
Hexameric Cluster. Inorg. Chem..

[179] Tian F., Chen J., Chen F., Liu Y., Xu Y., Chen R. (2021). Boosting Hydrogen Evolution over
Ni6­(SCH2Ph)­12 Nanocluster
Modified TiO2 via Pseudo-Z-Scheme Interfacial Charge Transfer. Appl. Catal. B Environ..

[180] Schubert J. S., Kalantari L., Lechner A., Giesriegl A., Nandan S. P., Alaya P., Kashiwaya S., Sauer M., Foelske A., Rosen J., Blaha P., Cherevan A., Eder D. (2021). Elucidating the Formation and Active
State of Cu Cocatalysts for Photocatalytic Hydrogen Evolution. J. Mater. Chem. A.

[181] Schubert J. S., Popovic J., Haselmann G. M., Nandan S. P., Wang J., Giesriegl A., Cherevan A. S., Eder D. (2019). Immobilization of Co,
Mn, Ni and
Fe Oxide Cocatalysts on TiO 2 for Photocatalytic Water Splitting Reactions. J. Mater. Chem. A.

[182] Dong J.-P., Xu Y., Zhang X.-G., Zhang H., Yao L., Wang R., Zang S.-Q. (2023). Copper-Sulfur-Nitrogen Cluster Providing
a Local Proton for Efficient Carbon Dioxide Photoreduction. Angew. Chem..

[183] Xie Z., Tan H. L., Wu H., Amal R., Scott J., Ng Y. H. (2022). Facet-Dependent
Spatial Charge Separation with Rational Cocatalyst
Deposition on BiVO4. Mater. Today Energy.

[184] Liu T., Pan Z., Vequizo J. J. M., Kato K., Wu B., Yamakata A., Katayama K., Chen B., Chu C., Domen K. (2022). Overall Photosynthesis
of H2O2 by an Inorganic Semiconductor. Nat.
Commun..

[185] Wang S., Li C., Qi Y., Zhang J., Wang N., Liu M., Zhang B., Cai X., Zhang H., Wei S., Ma G., Yang J., Chen S., Zhang F. (2025). Etched BiVO4 Photocatalyst
with Charge
Separation Efficiency Exceeding 90%. Nat. Commun..

[186] Botos A., Biskupek J., Chamberlain T. W., Rance G. A., Stoppiello C. T., Sloan J., Liu Z., Suenaga K., Kaiser U., Khlobystov A. N. (2016). Carbon
Nanotubes as Electrically Active Nanoreactors for Multi-Step Inorganic
Synthesis: Sequential Transformations of Molecules to Nanoclusters
and Nanoclusters to Nanoribbons. J. Am. Chem.
Soc..

[187] Zhong D., Sousa F. L., Müller A., Chi L., Fuchs H. (2011). A Nanosized Molybdenum Oxide Wheel with a Unique Electronic-Necklace
Structure: STM Study with Submolecular Resolution. Angew. Chem., Int. Ed..

[188] Kibsgaard J., Jaramillo T. F., Besenbacher F. (2014). Building an
Appropriate Active-Site Motif into a Hydrogen-Evolution Catalyst with
Thiomolybdate [Mo3S13]­2– Clusters. Nat.
Chem..

[189] Swierczewski M., Maroni P., Chenneviere A., Dadras M. M., Lee L.-T., Bürgi T. (2021). Deposition
of Extended Ordered Ultrathin Films of Au38­(SC2H4Ph)­24 Nanocluster
Using Langmuir–Blodgett Technique. Small.

[190] Sels A., Salassa G., Pollitt S., Guglieri C., Rupprechter G., Barrabés N., Bürgi T. (2017). Structural
Investigation of the Ligand Exchange Reaction with Rigid Dithiol on
Doped (Pt, Pd) Au25 Clusters. J. Phys. Chem.
C.

[191] Zimmerli N. K., Usuga A. F., Checchia S., Comas-Vives A., Müller C. R., Abdala P. M. (2025). How Does the Ni–Ga Alloy Structure
Tune Methanol Productivity and Selectivity?. ACS Catal..

[192] Larmier K., Liao W.-C., Tada S., Lam E., Verel R., Bansode A., Urakawa A., Comas-Vives A., Copéret C. (2017). CO2-to-Methanol Hydrogenation on Zirconia-Supported
Copper Nanoparticles: Reaction Intermediates and the Role of the Metal–Support
Interface. Angew. Chem., Int. Ed..

[193] Zhou H., Chen Z., López A. V., López E. D., Lam E., Tsoukalou A., Willinger E., Kuznetsov D. A., Mance D., Kierzkowska A., Donat F., Abdala P. M., Comas-Vives A., Copéret C., Fedorov A., Müller C. R. (2021). Engineering
the Cu/Mo2CTx (MXene) Interface to Drive CO2 Hydrogenation to Methanol. Nat. Catal..

[194] Qureshi M., Takanabe K. (2017). Insights on Measuring
and Reporting
Heterogeneous Photocatalysis: Efficiency Definitions and Setup Examples. Chem. Mater..

[195] Hoque M. A., Guzman M. I. (2018). Photocatalytic Activity: Experimental
Features to Report in Heterogeneous Photocatalysis. Materials.

[196] Kamat P. V., Jin S. (2018). Semiconductor Photocatalysis: “Tell
Us the Complete Story!”. ACS Energy Lett..

[197] Kramm U. I., Marschall R., Rose M. (2019). Pitfalls in Heterogeneous
Thermal, Electro- and Photocatalysis. ChemCatChem..

[198] Melchionna M., Fornasiero P. (2020). Updates on the Roadmap for Photocatalysis. ACS Catal..

[199] Cao S., Piao L. (2020). Considerations for
a More Accurate Evaluation Method
for Photocatalytic Water Splitting. Angew. Chem..

[200] Li W., He D., Sheehan S. W., He Y., Thorne J. E., Yao X., Brudvig G. W., Wang D. (2016). Comparison of Heterogenized Molecular
and Heterogeneous Oxide Catalysts for Photoelectrochemical Water Oxidation. Energy Environ. Sci..

[201] Wang Y., Li J., Hu Q., Hao M., Liu Y., Gong L., Li R., Huang X. (2021). Boosting Visible-Light-Driven
Photocatalytic Hydrogen Production through Sensitizing TiO2 via Novel
Nanoclusters. ACS Appl. Mater. Interfaces.

[202] Hao M., Hu Q., Zhang Y., Luo M., Wang Y., Hu B., Li J., Huang X. (2019). Soluble Supertetrahedral Chalcogenido
T4 Clusters: High Stability and Enhanced Hydrogen Evolution Activities. Inorg. Chem..

[203] Wu T., Wang L., Bu X., Chau V., Feng P. (2010). Largest Molecular
Clusters in the Supertetrahedral Tn Series. J. Am. Chem. Soc..

[204] Liu D., Fan X., Wang X., Hu D., Xue C., Liu Y., Wang Y., Zhu X., Guo J., Lin H., Li Y., Zhong J., Li D., Bu X., Feng P., Wu T. (2019). Cooperativity by Multi-Metals Confined
in Supertetrahedral Sulfide
Nanoclusters To Enhance Electrocatalytic Hydrogen Evolution. Chem. Mater..

