# Supplementary material for: Atomically Precise Cluster Cocatalysts: Missing Link toward Heterogenized Photocatalytic Systems
Source: ACS Nano. 2025 Dec 19;20(1):99–118. doi: 10.1021/acsnano.5c15286 (PMC12810487; doi:10.1021/acsnano.5c15286)
Supplement: Supplementary file 1 [file nn5c15286_si_001.pdf]

Electronic Supplementary Information

for

Atomically Precise Cluster Co-Catalysts: Missing Link Towards Heterogenized Photocatalytic Systems

Stephen Nagaraju Myakala and Alexey Cherevan*

Institute of Materials Chemistry, TU Wien, Getreidemarkt 9/BC/02, 1060, Vienna, Austria

Corresponding author

alexey.cherevan@tuwien.ac.at

Table S1. Summary table of various classes of co-catalysts on photoactive supports along with the reaction conditions and typical photocatalytic HER performance metrics reported (with AQY, TONs, and TOFs provided when available). Some of the activity values have been normalised based on the reported amounts of catalyst used to obtain performance comparability. References [1-10]* are extra works that have not been discussed in the main review article due to similarity with other examples mentioned.

Cluster	Support	Sacrificial agent	Illumination source	H_2 (μmol h^{-1} g^{-1})	AQY	TON/TOF	Year	Ref
$H_3PW_{12}O_{40}$	CdS – Au – NH_2	0.1M Na_2S/Na_2SO_3	500 W Xe lamp (>420)	550			2013	[113]
$[P_2W_{18}O_{62}]^{6-}$	UiO-Zr(Ru) MOF	10% aq. MeOH	visible light (>400)	49.9		79	2015	[116]
$Na_8Ta_6O_{19}$	$Cd_{0.7}Zn_{0.3}S$	0.35M Na_2S/Na_2SO_3	300 W Xe lamp (>420)	43050			2019	[112]
$[PW_{11}O_{39}\{cis\text{-}Pt(NH_3)_2\}_2]^{3-}$	TiO_2-APS	20% aq. MeOH	400 W Xe lamp (200-400 nm)	5600			2020	[111]
$K_{14}[O\{Re^V(OH)(\alpha_2\text{-}P_2W_{17}O_{61})\}_2]$	TiO_2-TMS	water vapour	500 W Xe lamp (>420)	3.96			2008	[107]
$[Ni_4(H_2O)_2(PW_9O_{34})_2]^{10-}$	CdS@CQDs	none	LED 100B (>420)	145		15.5 h^{-1}	2021	[**1**]*
$[Ni_4(H_2O)_2(PW_9O_{34})_2]^{10-}$ (homogeneous)	CdSe QDs	0.2M AA	LED 520 nm	6.6 μmolh^{-1}			2022	[**2**]*
$Na_6K_4[Ni_4(H_2O)_2(PW_9O_{34})_2]$	CdS@g-C_3N_4	0.35M Na_2S/Na_2SO_3	300 W Xe lamp (>420)	44000			2018	[**3**]*
$Na_{27}[Fe_{11}(H_2O)_{14}(OH)_2(W_3O_{10})_2(\alpha\text{-}SbW_9O_{33})_6]$	p-SiO_2-NH_2@CdS	10 vol% lactic acid	LED (100 mWcm^{-2}, >420nm)	23100	71% (420 nm)	3225	2022	[114]
$Na_6K_4[Ni_4(H_2O)_2(PW_9O_{34})_2]\cdot 32H_2O$	CdS	ACN/H_2O	300 W Xe lamp (>420)	649			2024	[**4**]*
$K_6Na[Ni_3(H_2O)_3PW_{10}O_{39}H_2O]$	NU-1000 MOF	1M AA	300 W Xe lamp (>420)	3482			2021	[117]
$Na_4Li_5[Ni_3(OH)_3(H_2O)_3P_2W_{16}O_{59}$	NU-1000 MOF	1M AA	300 W Xe lamp (>420)	13051		2724	2021	[117]
$[P_2W_{18}O_{62}]^{6-}$	SMOF	10% aq. MeOH or TEOA	visible light	888.2		1820 (14h)	2016	[**5**]*

$H_3PW_{12}O_{40}$	UiO-66	25% aq. MeOH	500 W Xe lamp (>420)	72.7			2018	[**6**]*
$Au_{25}(Cys)_{18}$	g-C_3N_4	10 vol% aq. TEOA	300 W Xe lamp (>420)	320	0.2% (420 nm)		2018	[**7**]*
$Au_{25}(SG)_{18}$	$BaLa_4Ti_4O_{15}$	none	high pressure Hg lamp	800			2013	[173]
$Au_{25}(PET)_{18}$	UiO-66-NH_2	10 wt% TEOA	300 W Xe lamp (>420)	17012			2023	[**8**]*
$[Ag_{44}(SePh)_{30}]^{4-}$	TiO_2 (P25)	20% aq. MeOH	MAX-302 Xenon lamp	1120			2023	[175]
$[Au_{12}Ag_{32}(SePh)_{30}]^{4-}$	TiO_2 (P25)	20% aq. MeOH	MAX-302 Xenon lamp	6810			2023	[175]
$Ni_6(SCH2Ph)_{12}$	TiO_2	20% aq. MeOH	300 W Xe lamp	5600			2021	[179]
$Ni_{12}(SPhCH_3)_{24}$	g-C_3N_4	20% aq. MeOH	300 W Xe lamp	3000			2021	[**9**]*
$[Mo_3S_7]^{4+}$	TiO_2	0.1 M Na_2S+0.02M Na_2SO_3	1000 W high-pressure Hg lamp	36		2.3 (8h)	2014	[80]
$[Mo_3S_{13}]^{2-}$	TiO_2	50 vol % aq. MeOH	LED 365 nm	6500			2022	[81]
$[Mo_2S_{12}]^{2-}$	TiO_2	15 vol % aq. MeOH	300 W Xenon lamp	213.1			2022	[82]
$[Mo_3S_{13}]^{2-}$	m-g-CN	10 vol% lactic acid	300W Xe lamp	116			2017	[83]
$[Mo_3S_{13}]^{2-}$	g-CN	0.1M TEOA	LED 445 nm	241		13.26 h^{-1}	2024	[84]
$[Mo_3S_{13}]^{2-}$	CdTe/CdS QDs (homogeneous)	20 mg/ml AA	300 W Xe lamp	150 μmolh^{-1}			2016	[**10**]*
$[Mo_3S_{13}]^{2-}$	p-CN	10 vol% aq. MeOH	LED 420 nm	166	0.055% (EQE)		2020	[85]

Abbreviations: aq. MeOH – aqueous methanol; AA – ascorbic acid; ACN – acetonitrile; TEOA – triethanolamine; UiO-Zr(Ru) – UiO-based MOF made from Ru-modified ligand and $ZrCl_4$; APS – aminopropyl silane; TMS – trimethoxy silane; CQDs – carbon quantum dots; Cys – L-cysteine; Ph – Phenyl; SMOF – Supramolecular metal-organic frameworks; p-CN – polymeric carbon nitride; g-CN – graphitic carbon nitride; m-g-CN – modified g-CN; EQE – external quantum efficiency.

References:

(1) Dong, Y.; Han, Q.; Hu, Q.; Xu, C.; Dong, C.; Peng, Y.; Ding, Y.; Lan, Y. Carbon Quantum Dots Enriching Molecular Nickel Polyoxometalate over CdS Semiconductor for Photocatalytic Water Splitting. *Appl. Catal. B Environ.* **2021**, *293*, 120214. https://doi.org/10.1016/j.apcatb.2021.120214.

(2) Zhang, M.; Xin, X.; Feng, Y.; Zhang, J.; Lv, H.; Yang, G.-Y. Coupling Ni-Substituted Polyoxometalate Catalysts with Water-Soluble CdSe Quantum Dots for Ultraefficient Photogeneration of Hydrogen under Visible Light. *Appl. Catal. B Environ.* **2022**, *303*, 120893. https://doi.org/10.1016/j.apcatb.2021.120893.

(3) Zhai, X.-L.; Liu, J.; Hu, L.-Y.; Bao, J.-C.; Lan, Y.-Q. Polyoxometalate-Decorated g-C3N4-Wrapping Snowflake-Like CdS Nanocrystal for Enhanced Photocatalytic Hydrogen Evolution. *Chem. – Eur. J.* **2018**, *24* (59), 15930–15936. https://doi.org/10.1002/chem.201803621.

(4) Ren, M.; Liu, T.; Dong, Y.; Li, Z.; Yang, J.; Diao, Z.; Lv, H.; Yang, G.-Y. Near-Unity Photocatalytic Dehydrocoupling of Thiophenols into Disulfides and Hydrogen Using Coupled CdS Nanorods and Ni-Containing Polyoxometalate. *Chin. J. Catal.* **2024**, *61*, 312–321. https://doi.org/10.1016/S1872-2067(24)60025-2.

(5) Tian, J.; Xu, Z.-Y.; Zhang, D.-W.; Wang, H.; Xie, S.-H.; Xu, D.-W.; Ren, Y.-H.; Wang, H.; Liu, Y.; Li, Z.-T. Supramolecular Metal-Organic Frameworks That Display High Homogeneous and Heterogeneous Photocatalytic Activity for H2 Production. *Nat. Commun.* **2016**, *7* (1), 11580. https://doi.org/10.1038/ncomms11580.

(6) Tian, P.; He, X.; Li, W.; Zhao, L.; Fang, W.; Chen, H.; Zhang, F.; Zhang, W.; Wang, W. Zr-MOFs Based on Keggin-Type Polyoxometalates for Photocatalytic Hydrogen Production. *J. Mater. Sci.* **2018**, *53* (17), 12016–12029. https://doi.org/10.1007/s10853-018-2476-0.

(7) Wang, C.; Lv, P.; Xue, D.; Cai, Y.; Yan, X.; Xu, L.; Fang, J.; Yang, Y. Zero-Dimensional/Two-Dimensional Au25(Cys)18 Nanoclusters/g-C3N4 Nanosheets Composites for Enhanced Photocatalytic Hydrogen Production under Visible Light. *ACS Sustain. Chem. Eng.* **2018**, *6* (7), 8447–8457. https://doi.org/10.1021/acssuschemeng.8b00643.

(8) Yao, A.; Du, Y.; Han, M.; Wang, Y.; Hu, J.; Zhu, Q.; Sheng, H.; Zhu, M. Covalence Bridge Atomically Precise Metal Nanocluster and Metal-Organic Frameworks for Enhanced Photostability and Photocatalysis. *Nano Res.* **2023**, *16* (1), 1527–1532. https://doi.org/10.1007/s12274-022-4725-4.

(9) Tian, F.; Huang, X.; Li, W.; An, Y.; Li, G.; Chen, R. Weak Interaction between Nickel Thiolate and G-C3N4 Improving Electron–Hole Separation for Photocatalysis. *ACS Catal.* **2023**, *13* (18), 12186–12196. https://doi.org/10.1021/acscatal.3c03063.

(10) Yue, D.; Qian, X.; Zhang, Z.; Kan, M.; Ren, M.; Zhao, Y. CdTe/CdS Core/Shell Quantum Dots Cocatalyzed by Sulfur Tolerant [Mo3S13]2– Nanoclusters for Efficient Visible-Light-Driven Hydrogen Evolution. *ACS Sustainable Chem. Eng.* **2016**, *4*, 12, 6653–6658. https://doi.org/10.1021/acssuschemeng.6b01520
